# Shenkang Injection for Treating Renal Fibrosis-Metabonomics and Regulation of E3 Ubiquitin Ligase Smurfs on TGF-β/Smads Signal Transduction

**DOI:** 10.3389/fphar.2022.849832

**Published:** 2022-06-02

**Authors:** Junju Zou, Xiaotao Zhou, Xian Chen, Yuerong Ma, Rong Yu

**Affiliations:** ^1^ Hunan Provincial Key Laboratory of Translational Research in TCM Prescriptions and Zheng, Hunan University of Chinese Medicine, Changsha, China; ^2^ School of Basic Medicine, Chengdu University of Chinese Medicine, Chengdu, China; ^3^ Hospital of Chengdu University of Traditional Chinese Medicine, Chengdu, China

**Keywords:** chronic kidney disease, transforming growth factor-β, Shenkang injection, TGF-β/Smad signaling pathway, ubiquitination, metabonomics

## Abstract

At present, TGF-β is the most critical fibrogenic factor known. Smad ubiquitin ligase Smurfs play an important role in the regulation of the TGF-/Smads signaling pathway, which is linked to metabolite changes in renal fibrosis. Previous studies have shown that Shenkang injection can prevent and treat chronic kidney disease through multiple channels of action. However, the precise relationship between Shenkang injection and the regulation of the TGF-/Smads signaling pathway in the treatment of chronic kidney disease is unknown. Here, we evaluated the pharmacological effects of Shenkang injection on ubiquitination and metabolic changes of the TGF-β/Smads signaling pathway in UUO mice using pathology-related indicators, immunoprecipitation, subcellular co-location, and metabonomics analysis. Our findings indicate that Shenkang injection can promote nuclear translocation of Smurf1 and Smurf2 to TGF- membrane receptors TR-I and Smad2 and ubiquitinated degradation of these proteins. Furthermore, the formation of TβR-I/TβR-II, TβR-I/Smad2, and TβR-I/Smad3 complexes was inhibited to negatively regulate the TGF-β/Smad signaling pathway induced renal tubular epithelial transdifferentiation (EMT). The EMT process is not very relevant *in vivo*, although it is clear that TGF-β induces EMT in cultured cells, which has been demonstrated by numerous teams around the world. However, this is not the case with the *in vivo* models of kidney fibrosis, especially UUO. In addition, Shenkang injection can improve amino acid metabolism, purine metabolism, and fatty acid metabolism disorders.

## 1 Highlights

1) Shenkang injection upregulated the expression of E-Cadherin, downregulated the expression of α-SMA and Collagen-I, and inhibited the EMT of renal interstitial cells in UUO mice.
2) Shenkang injection can upregulate the expression of Smurf1, Smurf2, Smad7 protein, and mRNA to inhibit EMT of renal interstitial cells.
3) Shenkang injection inhibited smad2/3 phosphorylation by selectively decreasing the interaction of TβR-II/TβR-I, TβR-I/Smad2, and TβR-I/Smad3 in HK-2 cells induced by TGF-β1, negatively regulating the TGF-β/Smads signaling pathway.
4) Shenkang injection can regulate the metabolism of amino acid, purine, and fatty acids in UUO mice.

## 2 Introduction

Renal fibrosis is a complex and irreversible pathological process that involves the activation and interaction of multiple pro-fibrotic signaling pathways. It is a late-stage feature of all types of chronic kidney disease (CKD), affecting more than 10% of the world’s population and posing a major public health challenge (Luyckx et al., 2020). Without alternative treatment, such as dialysis or kidney transplantation, CKD can progress to end-stage renal disease (Zhu et al., 2020). Although scientists have repeatedly studied the idea of reversing CKD over the past decades, existing treatments to prevent CKD progression and CKD-related complications are quite limited (Holden et al., 2020). Current interventions include angiotensin-converting enzyme inhibition, angiotensin receptor block, optimal blood pressure control, and sodium bicarbonate for metabolic acidosis (Onuigbo and Agbasi, 2015). However, none of these treatments improved renal function, and the patient maintained poor renal function. Therefore, it is urgent to find effective drugs to treat CKD.

TGF-β/Smads signaling is the primary pathway of fibrosis formation, according to numerous studies (Kahata et al., 2018). Activation of TGF-β/Smads signals leads to extracellular matrix synthesis and deposition, podocyte depletion, mesangial dilation, renal tubular epithelial fibrosis transformation, and myoblast fibroblast activation. TGF-β1 activation recruits and activates type II TGF receptors (TβRII) and downstream receptor-associated Smads (R-Smads), Smad2, and Smad3. Phosphorylated Smad2/3 then forms oligomeric complexes with Smad4 (Derynck and Zhang, 2003; Lan and Chung, 2012). Subsequently, the Smad2/3/4 complex is translocated to the nucleus to regulate the transcription of target genes and induce α -smooth muscle actin (α-SMA), Collagen I, and inhibitory Smad7 (Nakao et al., 1997; Miyazawa and Miyazono, 2017). Interestingly, Smad7 antagonizes a variety of diseases, including TGF-β-mediated fibrosis, cancer, and inflammation (Yan et al., 2009; Troncone et al., 2018; Zhou et al., 2018). Smad7 negatively regulates TGF-β/Smad signaling by competing with R-smad and binding to TβRI (Yan et al., 2016). Ubiquitin-mediated proteasome degradation pathway is an evolutionarily conserved cascade that strictly regulates TGF-β superfamily signal transduction (Nakamura, 2018). Smad ubiquitin regulatory factor 1 (Smurf1) and Smurf2 are HECT (homologous to the C-terminal of E6 co-protein) E3 ubiquitin ligases that regulate TGF-β and BMP signaling (Zhu et al., 1999; Kavsak et al., 2000; Lin et al., 2000; Ebisawa et al., 2001). Smad7 recruits Smurf1 and Smurf2, forms a complex with Smurfs, translocates from the nucleus to TGF-β membrane receptors TβR-I and Smad2, and degrades the complex through the proteasome pathway (Lin et al., 2000; Ebisawa et al., 2001). These studies indicate that TGF-β/Smads signaling plays a major role in renal fibrosis (Yan et al., 2009; Troncone et al., 2018; Zhou et al., 2018). Smad7 negatively regulates TGF-β/Smad signaling by competing with R-smad and binding to TβRI (Yan et al., 2016). The ubiquitin-mediated proteasome degradation pathway is an evolutionarily conserved cascade that tightly controls TGF-superfamily signal transduction (Nakamura, 2018). Smad ubiquitin regulatory factor 1 (Smurf1) and Smurf2 are HECT (homologous to the C-terminus of E6 co-protein) E3 ubiquitin ligases that control TGF and BMP signaling (Zhu et al., 1999; Kavsak et al., 2000; Lin et al., 2000; Ebisawa et al., 2001). Smad7 recruits Smurf1 and Smurf2, forms a complex with Smurfs, translocates from the nucleus to the TGF membrane receptors TR-I and Smad2, and degrades the complex *via* the proteasome pathway (Lin et al., 2000; Ebisawa et al., 2001). These findings suggest that TGF-/Smad signaling is important in renal fibrosis.

Studies have shown that the TGF-β/Smads signaling pathway is closely related to metabolic disorders. TGF-β/Smads signaling regulates the expression of genes involved in fat formation and fatty acid β-oxidation, resulting in increased triglyceride synthesis and lipid accumulation in hepatocytes (Tan et al., 2011). It also regulates the expression of genes involved in fat formation and fatty acid oxidation (Yang et al., 2014). Up-regulated expression of extracellular matrix (ECM) components TGF-β1, connective tissue growth factor (CTGF), fibroblastic growth factor (bFGF), and collagen I was observed in patients with hepatic fibrosis, accompanied by changes in lipid metabolism, amino acid metabolism, purine metabolism, and taurine metabolism (Bianchi et al., 2000; Zhang et al., 2006; Zhao et al., 2014). Therefore, the transmission of the TGF-β/Smads signal pathway is closely related to the disorder of lipid metabolism pathway, amino acid metabolism pathway, and purine metabolism pathway, and the regulation of TGF-β/Smads signal transduction can improve the level of disordered metabolites.

Shenkang injection (SKI) is one of the representative Chinese patent medicine preparations. SKI is developed by renowned and experienced Chinese medicine practitioners and consists of four herbal extracts: Radix Et Rhizoma Rhei Palmati, Radix Astragali Mongolici, HerbaSalviae Japonicae, and Flos Carthami. SKI, as a modern proprietary Chinese medicine intravenous injection (Z20040110), was approved to be marketed by the State Food and Drug Administration of China in 2004. After more than ten years of clinical use, certain clinical research evidence has been accumulated, and it can be used to intervene in strengthening factors, cytokines, chemokines, and fibrosis related pathways (Zou et al., 2020), control the inflammatory response (Derynck and Zhang, 2003), alleviate renal oxidative stress (Ebisawa et al., 2001), improve glomerular filtration (Kavsak et al., 2000), and prevent glomerulosclerosis (Derynck and Zhang, 2003), so as to achieve the effect of chronic kidney disease.

It is unclear whether SKI can improve metabolic disorders in UUO mice or promote ubiquitination and degradation of the TGF-/Smad signaling pathway. In this study, the anti-fibrosis, metabolite alteration, and EMT phenomena of SKI were evaluated in HK-2 cells and UUO mice, with molecular mechanism studies focusing on ubiquitination of the TGF-β/Smad signaling pathway.

## 3 Materials and Methods

### 3.1 Reagents

SKI was purchased from Xi’an Century Shenkang Pharmaceutical Industry Co., Ltd. (Xi’an, China, 202102103). TGF-β1 (Santa Cruz, sc-130348), E-Cad (CST, #3195), P-Smad3 (Abclonal, A19115), P-Smad2/3 (Abclonal, A19115), Smurf2 (Santa Cruz, sc-518164), α-SMA (CST, #19245), collagen I (Abclonal, A5786), Smad7 (Santa Cruz, sc-365846), Smad2/3 (Santa Cruz, sc-133098), ubiquitin (Santa Cruz, sc-8017), TβR-I (Santa Cruz, sc-101574), TβR-II (Santa Cruz, sc-1700), Smad3 (Santa Cruz, sc-101154), serum creatinine (Scr), and blood urea nitrogen (BUN) assay kits were obtained from Nanjing Jiancheng Bioengineering Institute (Nanjing, China). Real-Time PCR Easy™- SYBR Green I (FOREGENE, QP-01014).

### 3.2 Chemical Analysis of CKI

**FIGURE 1:**
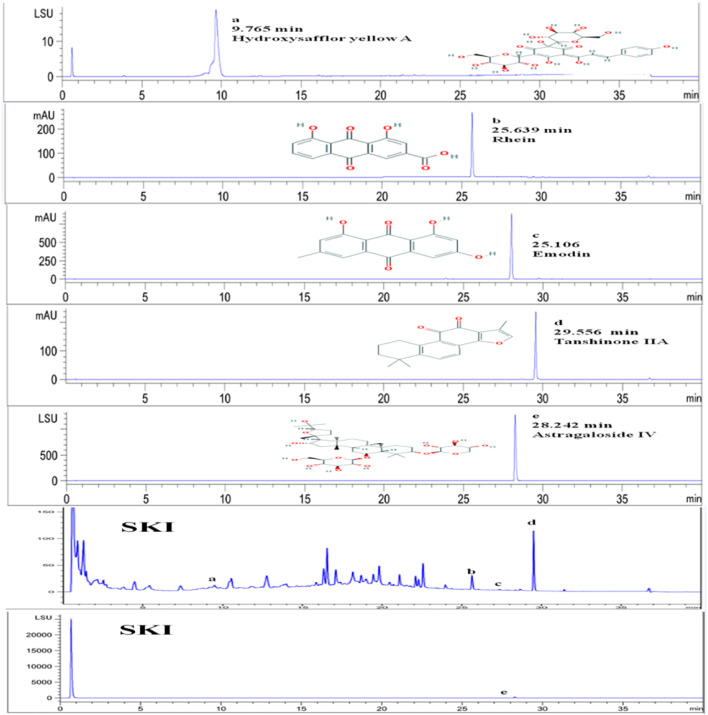
Qualitative analysis of five main effective components of Shenkang injection.

SKI is composed of extracts from a defined mixture of Chinese herbs as follows, radix et rhizoma rhei (Rheum palmatum L. Dahuang), radix astragali [*Astragalus* membranaceus (Fisch.) Beg. Huangqi], radix salviae miltiorrhizae (Salvia miltiorrhiza Bunge. Danshen), and Flos carthami (Carthamus tinctorius L. Honghua). One injection (20 ml) contains 6 g of the abovementioned extracts. The extracted method and the productive process of SKI, protected by the patent law of China, are both subjected to strict quality control, and the main components are subjected to standardization. The quality of SKI was measured with fingerprint analysis by HPLC based on the report by Xu et al. (2017) and Yao et al. (2015). To assure the consistency of SKI, an accurate and practical ultra-performance liquid chromatography (UPLC) method based on the Chinese Pharmacopoeia was used for chromatographic fingerprint analysis of SKI and the simultaneous determination of five active constituents (hydroxy safflor yellow A, astragaloside IV, rhein, tanshinone IIA, and emodin) in SKI (Figure 1).

### 3.3 Animals

C57BL/6J mice (male, 6–8 weeks) were purchased from the Center for Chengdu Dasuo Experimental Animal Co., Ltd. (license key: SCXK (Chuan) 2015–030, Chengdu, China). All mice were maintained in cages (4–6 mice/cage) under a standard light/dark cycle (12:12/h) with free access to food and water ad libitum. All experimental animals were allowed to acclimatize for a period of 1 week before the initiation of the experiment. The animal experiments were approved by the Committee of Scientific Research and the Committee of Animal Care of the Chengdu University of Traditional Chinese Medicine (Chengdu, China), and all procedures were conducted in accordance with the Helsinki Declaration. After 1 week of adaptive feeding, the mice were randomly divided into five groups (*n* = 6/group), as follows: the sham operation group (Sham), the UUO group (UUO), the UUO+10 mg/kg/d Losartan treatment group (ARB), and the UUO+(0.15, 0.3, 0.6 ml/20 g/d) SKI treatment group (SKI-L, SKI-M, and SKI-H).

As previously described, a mouse model of UUO nephropathy was established by ligation of the left ureter (Chevalier et al., 2009). On day 1, sodium pentobarbital (75 mg/kg) was given for abdominal anesthesia. The left ureter was separated through a midline abdominal incision and ligated with a 4-0 wire. In the Sham, the ureter was free and not ligated. The dosages of losartan and Shenkang injection were selected based on previous reports. On day 4, after obstructive surgery, mice were intragastric with losartan (Yao et al., 2018), and an intraperitoneal injection of SKI (Liu Y. et al., 2019) was administered once a day for 14 consecutive days. All mice were sacrificed 14 days after the operation under pentobarbital sodium anesthesia.

### 3.4 Cell Culture and Treatment

Human kidney-2 (HK-2) cells were obtained from the Chinese Academy of Sciences Cell Bank (Shanghai, China). Cells were cultured in a DMEM medium containing 10% FBS in an incubator at 37°C. For the detection of cell transdifferentiation, the HK-2 cells were treated with 5, 10, and 20 ng/ml TGF-β1 for 48h or 1:40, 1:80, and 1:100 SKI plus 10 ng/ml TGF-β1 for 48 h.

### 3.5 Cell Viability Assay

HK-2 cells (2 × 10^3^ cells/well) were placed into 96-well plates, and treated with 1:10, 1:20, 1:40, 1:80, 1:100, 1:200, 1:400, 1:800, 1:1,000, 1:10,000, and 1:100,000 SKI for 24, 48, and 72 h. The culture supernatant was removed, and then CCK-8 was added to the wells for 0.5–1 h. Finally, the plate reader was used to detect the absorbance at 450 nm.

### 3.6 Immunofluorescence Staining

Immunofluorescence staining was performed using established procedures (Li et al., 2019). Corresponding drugs and reagents were given according to the groups for 48 h intervention. After 48 h, all liquid was sucked away, cells were cleaned with PBS for 3 times, PBS was sucked away, and 4% paraformaldehyde was added to fix for 20 min. The fixative solution was removed and rinsed with PBS for 3 times. After being sealed with 5%BSA at room temperature for 1 h, the primary antibody was added at 4°C overnight. After washing with PBS for 3 times, the corresponding secondary antibody was added, and the cells were incubated at room temperature without light for 1 h. Then, DAPI was added for nuclear staining, and the cells were incubated at room temperature without light for 20 min. After washing, the tablets were sealed with 50% glycerol solution. The cover glass was observed using a Leica confocal microscope.

### 3.7 Immunohistochemistry

As mentioned earlier, we use the principle of specific binding of antigen and antibody to perform immunohistochemistry experiments (Lv et al., 2019). Related antibodies are as follows: E-Cad (1:200), P-Smad2/3 (1:200), Smurf1, Smurf2, α-SMA (1:200), vimentin (1:200), Smad7 (1:200), TβR-I (1:200), TβR-II (1:200), and ubiquitin (1:200).

### 3.8 Western Blot Analysis

Cells and mouse kidney tissue were lysed with RIPA lysis buffer, as described previously (Han et al., 2021). Collect cell samples to lyse, centrifuge to extract protein, use BCA kit for protein quantification, perform SDS-PAGE gel electrophoresis and transfer membrane, block with 5% skim milk at room temperature for 1 h, add the corresponding primary antibody overnight at 4°C, after overnight rinsing with TBST 3 times and adding the corresponding HRP IgG, incubate at room temperature for 1 h, wash with TBST 3 times, and finally exposed to ECL solution and photographed for retention. ImageJ software was used for grayscale analysis, β-actin was used as an internal reference, and the ratio of the target protein to β-actin was used to indicate the relative expression of the target protein.

### 3.9 RT-PCR

Total RNA was extracted using the total RNA isolation kit (Foregene, Chengdu, China) according to the manufacturer’s instructions (Lv et al., 2019). After reverse transcription, the cDNAs were amplified on a qTOWER3/qTOWER3 touch Real-Time PCR System (Analytik Jena) under the following conditions: initial denaturation for 3 min at 95°C followed by 40 cycles of denaturation at 95°C for 5 s, annealing, and extension at 65°C for 30 s. GAPDH was used for normalization. The results were analyzed using the 2^−ΔΔCt^ method. The sequences of the primers are given in Tables 1 and 2.

**TABLE 1:** Primer sequences for quantitative real-time PCR amplification (Mouse).

Gene	Forward primers (5–3′)	Reverse primer (5–3′)
GAPDH	TGA​CCT​CAA​CTA​CAT​GGT​CTA​CA	CTT​CCC​ATT​CTC​GGC​CTT​G
α-SMA	GGC​ACC​ACT​GAA​CCC​TAA​GG	ACA​ATA​CCA​GTT​GTA​CGT​CCA​GA
E-Cadherin	TCG​GAA​GAC​TCC​CGA​TTC​AAA	CGG​ACG​AGG​AAA​CTG​GTC​TC
TGF-β1	GAG​CCC​GAA​GCG​GAC​TAC​TA	TGG​TTT​TCT​CAT​AGA​TGG​CGT​TG
COL-I	TAA​GGG​TCC​CCA​ATG​GTG​AGA	GGG​TCC​CTC​GAC​TCC​TAC​AT
Smad3	CAT​TCC​ATT​CCC​GAG​AAC​ACT​AA	GCT​GTG​GTT​CAT​CTG​GTG​GT
Smad7	GGG​CTT​TCA​GAT​TCC​CAA​CTT	AGG​GCT​CTT​GGA​CAC​AGT​AGA
TβR-I	AAA​ACA​GGG​GCA​GTT​ACT​ACA​AC	TGG​CAG​ATA​TAG​ACC​ATC​AGC​A
TβR-II	AAC​ATG​GAA​GAG​TGC​AAC​GAT	CGT​CAC​TTG​GAT​AAT​GAC​CAA​CA
Smurf1	AGC​ATC​AAG​ATC​CGT​CTG​ACA	CCA​GAG​CCG​TCC​ACA​ACA​AT
Smurf2	CCA​TTT​GCT​AAG​GTG​GTA​GTT​GA	CAG​GTC​ATA​ATG​CTG​ATT​CCA​CT

**TABLE 2:** Primer sequences for quantitative real-time PCR amplification (Human).

Gene	Forward primers (5–3′)	Reverse primer (5–3′)
GAPDH	ACA​ACT​TTG​GTA​TCG​TGG​AAG​G	GCC​ATC​ACG​CCA​CAG​TTT​C
α-SMA	GTG​TTG​CCC​CTG​AAG​AGC​AT	GCT​GGG​ACA​TTG​AAA​GTC​TCA
E-Cadherin	ATT​TTT​CCC​TCG​ACA​CCC​GAT	TCC​CAG​GCG​TAG​ACC​AAG​A
TGF-β1	CTA​ATG​GTG​GAA​ACC​CAC​AAC​G	TAT​CGC​CAG​GAA​TTG​TTG​CTG
COL-I	GTG​CGA​TGA​CGT​GAT​CTG​TGA	CGG​TGG​TTT​CTT​GGT​CGG​T
Smad3	CCA​TCT​CCT​ACT​ACG​AGC​TGA​A	CAC​TGC​TGC​ATT​CCT​GTT​GAC
Smad7	GGA​CAG​CTC​AAT​TCG​GAC​AAC	GTA​CAC​CCA​CAC​ACC​ATC​CAC
TβR-I	GCT​GTA​TTG​CAG​ACT​TAG​GAC​TG	TTT​TTG​TTC​CCA​CTC​TGT​GGT​T
TβR-II	AAG​ATG​ACC​GCT​CTG​ACA​TCA	CTT​ATA​GAC​CTC​AGC​AAA​GCG​AC
Smurf1	TGT​GAA​AAA​CAC​ATT​GGA​CCC​A	ACG​CTA​ATG​GTT​ATC​GAA​TCC​G
Smurf2	CGG​TTG​TGT​TCG​TCT​TCT​TTC​C	GCC​CGA​GTT​TGC​ATA​AAT​CCA

### 3.10 Sample Preparation and UPLC-Q-TOF-MS/MS Analysis for Metabolomics

Use non-targeted metabolomics UPLC-Q-TOF-MS/MS for plasma metabolite analysis. The metabolic process, including sample preparation, metabolite separation and detection, data preprocessing, and statistical analysis for metabolite identification, is slightly modified on this basis (Chen et al., 2017; Hoel et al., 2021).

### 3.11 Statistical Analysis

Data are expressed as mean ± standard deviation (SD). Nonparametric Kruskal–Wallis one-way analysis of variance (ANOVA) was used to compare the ranking data between different groups. When the difference is significant (*p* < 0.05), the Mann–Whitney *U* test is performed, which is determined by pairwise comparison of significant differences in each group.

## 4 Results

### 4.1 Detection of the Main Active Ingredients in Shenkang Injection With UPLC System

The five effective components of hydroxysafflor yellow A, astragaloside IV, rhein, tanshinone ⅡA, and emodin in Shenkang injection were qualitatively analyzed. The test found that the Shenkang injection contained hydroxysafflor yellow A, astragaloside IV, rhein, tanshinone IIA, and emodin (Figure 1).

### 4.2 Effect of Shenkang Injection on Body Weight, Kidney, SCr, and BUN of UUO Mice

**FIGURE 2:**
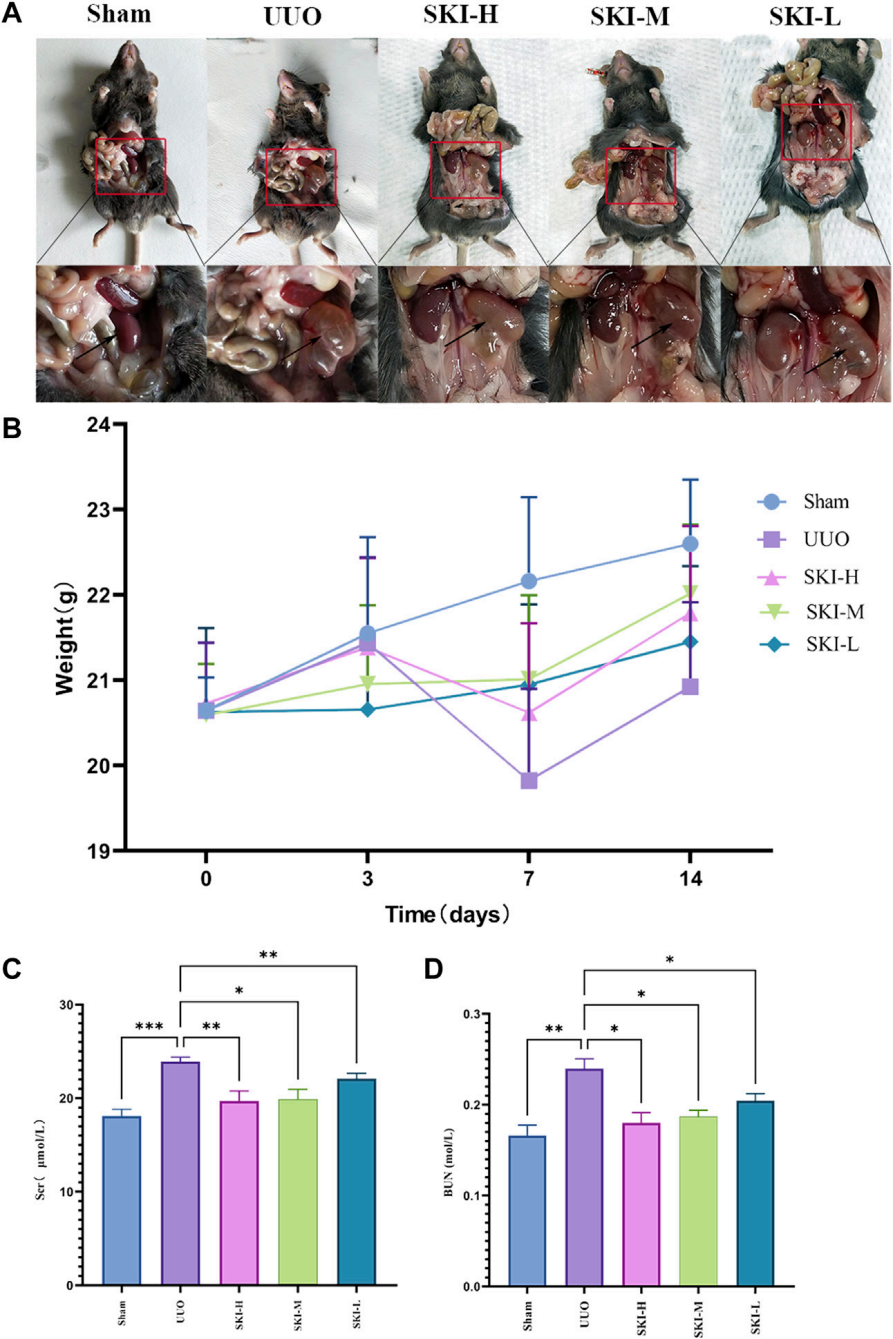
Effects of Shenkang injection on body weight, Scr, and BUN of UUO model mice. **(A)** Renal effusion on the ligation side. **(B)** Changes in body weight of mice. **(C)** and **(D)** Changes of Scr and BUN in mice. Compared with the model group, *p < 0.05 and **p < 0.01.

UUO mice were treated with Shenkang Injection for 14 days. It was observed that the renal effusion on the affected side of the drug group decreased significantly (Figure 2A). Compared with the Sham group, the weight of mice in the UUO group decreased significantly from the seventh day. Compared with the UUO group, the weight of each treatment group of Shenkang injection increased (*p* < 0.05) (Figure 2B). The difference was statistically significant. Compared with the Sham, the expressions of Scr and BUN in the UUO group were increased (*p* < 0.05) (Figure 2C). Compared with the UUO group, the expression of SCR and BUN in each treatment group of Shenkang injection showed a gradient downward trend (*p* < 0.05) (Figure 2C).

### 4.2 Effect of Shenkang Injection on Histopathology of UUO Mice

**FIGURE 3:**
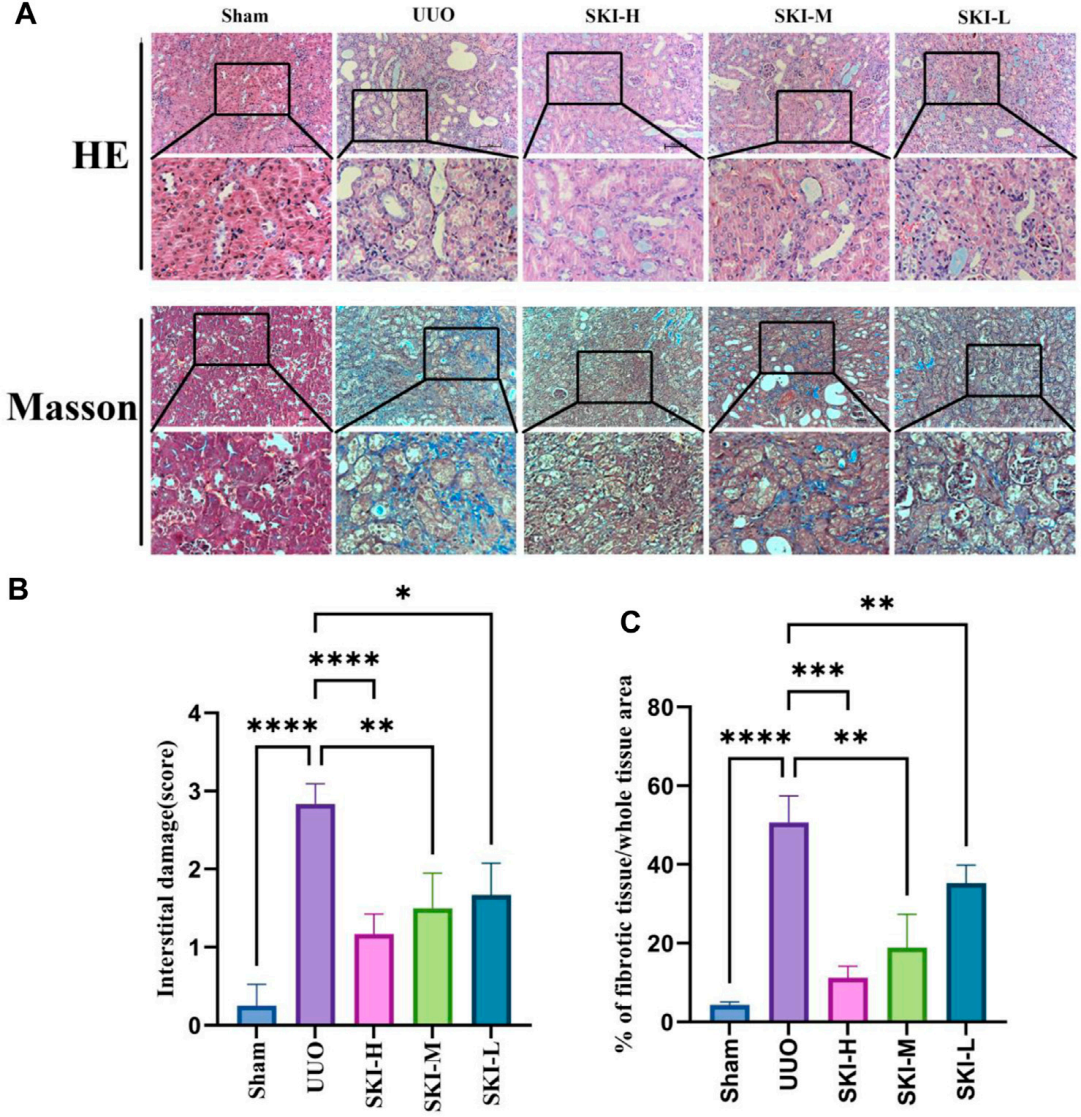
Effect of Shenkang injection on kidney histological changes and collagen deposition in UUO mice (200X). **(A)** HE staining and Masson staining in different groups, original magnification: ×200. **(B** and **C)** The degree of tubulointerstitial injury and tubulointerstitial collagen deposition were evaluated semi-quantitatively. Data are shown as mean ± SD. *p < 0.05, **p < 0.01, ***p < 0.001, and ****p < 0.0001.

After 14 days of modeling, the Sham did not show any pathological morphological changes. The HE staining results of the UUO showed severe renal tubular atrophy and dilation, a decrease in the number of renal tubules, cystic dilated renal tubular epithelial cell apoptosis, slight inflammatory cell infiltration, and obvious interstitial fibrosis. Compared with the UUO, these injuries were reduced to varying degrees in each treatment group of Shenkang injection. The results of Masson staining showed that UUO caused a large number of collagen fibers to accumulate in the renal tubular interstitium of UUO mice, and each treatment group of Shenkang injection reduced the area of renal tubular interstitial fibrosis (*p* < 0.0001). The result is shown in Figure 3.

### 4.3 Effect of Shenkang Injection on ECM Deposition and EMT Formation in Mouse Kidney

**FIGURE 4:**
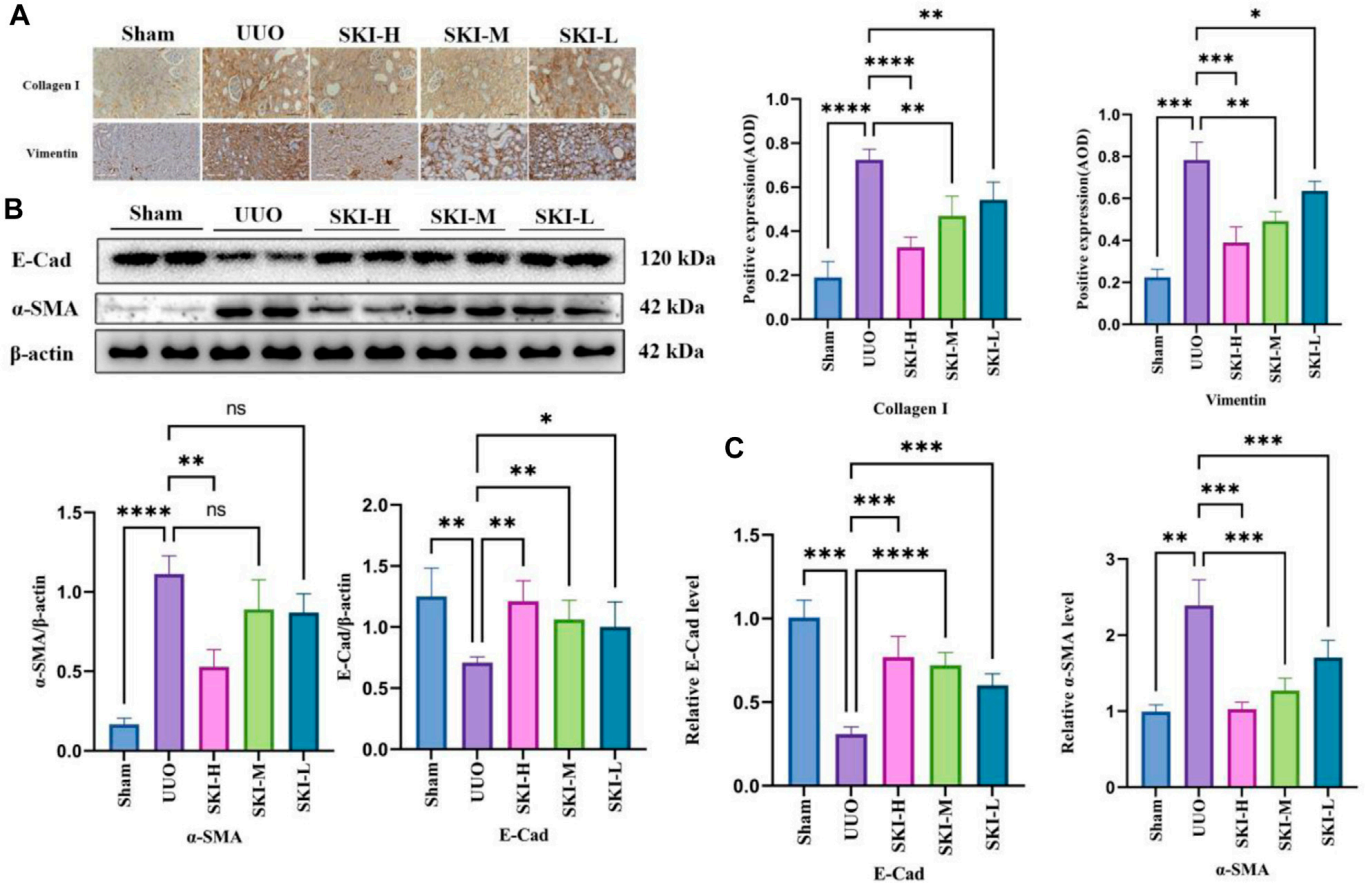
Effects of Shenkang injection on ECM deposition and EMT formation in the kidney of UUO mice. **(A)** The expression of COL-I and vimentin protein in the kidney tissue sections by immunohistochemistry, scale bar, 100 μm. **(B)** The expression of α-SMA and E-Cad in renal tissue lysates by Western blot and quantified by densitometry. **(C)** The expression of α-SMA and E-Cad mRNA in kidney tissue by RT-PCR.

In order to evaluate the effect of Shenkang injection on UUO induced fibrosis, we analyzed the deposition of several ECMs, including collagen I, collagen III, and vimentin. UUO’s kidneys had higher levels of collagen I, collagen III, and vimentin (P 0.05), indicating that UUO induced renal interstitial fibrosis over time. Shenkang injection treatment can alleviate the deposition of all these ECMs (*p* < 0.01). Transdifferentiation of renal tubular epithelial cells is essential for renal interstitial fibrosis. Transdifferentiated renal tubular epithelial cells are the source of ECM. The staining of α-SMA, E-Cad, and vimentin by immunohistochemistry showed that compared with the Sham, the expression of E-Cad protein in the kidney of UUO decreased, and the expression of α-SMA and vimentin protein increased (*p* < 0.05). Compared with the UUO, the expression of α-SMA and vimentin protein in each treatment group of Shenkang injection decreased, and the expression of E-Cad increased (*p* < 0.05) (Figure 4).

### 4.4 Effect of Shenkang Injection on TGF-β/Smads Signal Pathway

**FIGURE 5:**
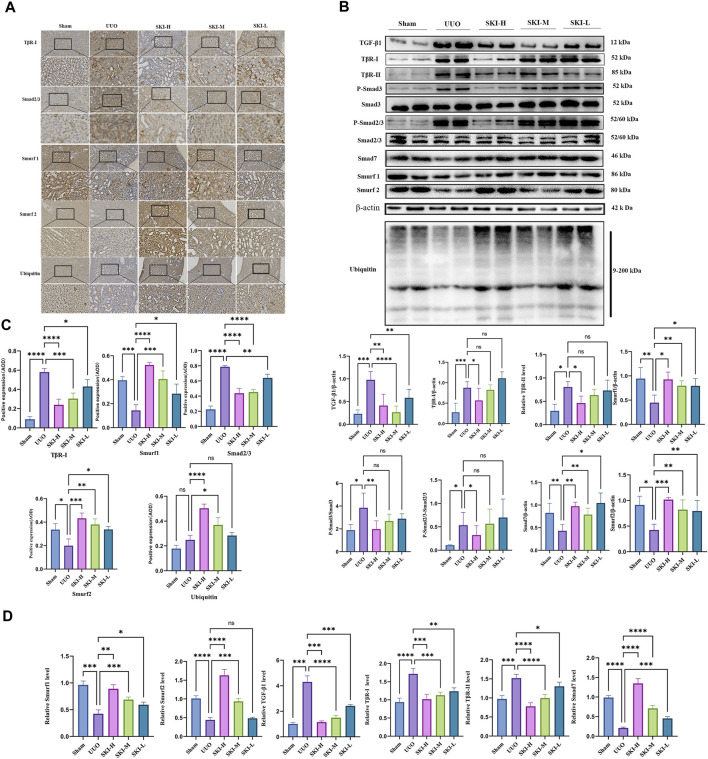
Effect of Shenkang injection on protein of TGF-β/Smads signaling pathway. **(A)** TβR-I, Smad2/3, Smurf1, Smurf2, and ubiquitin protein expression, Scale: 100 um (200×). **(B)** Western blotting of Smad7, P-Smad3, P-Smad2/3, TβR-I, TβR-II, Smurf1, Smurf2, and ubiquitin. **(C)** The average optical density (AOD) in kidney tissue sections by immunohistochemistry. **(D)** Mean optical density of renal tissue protein (AOD).

**FIGURE 6:**
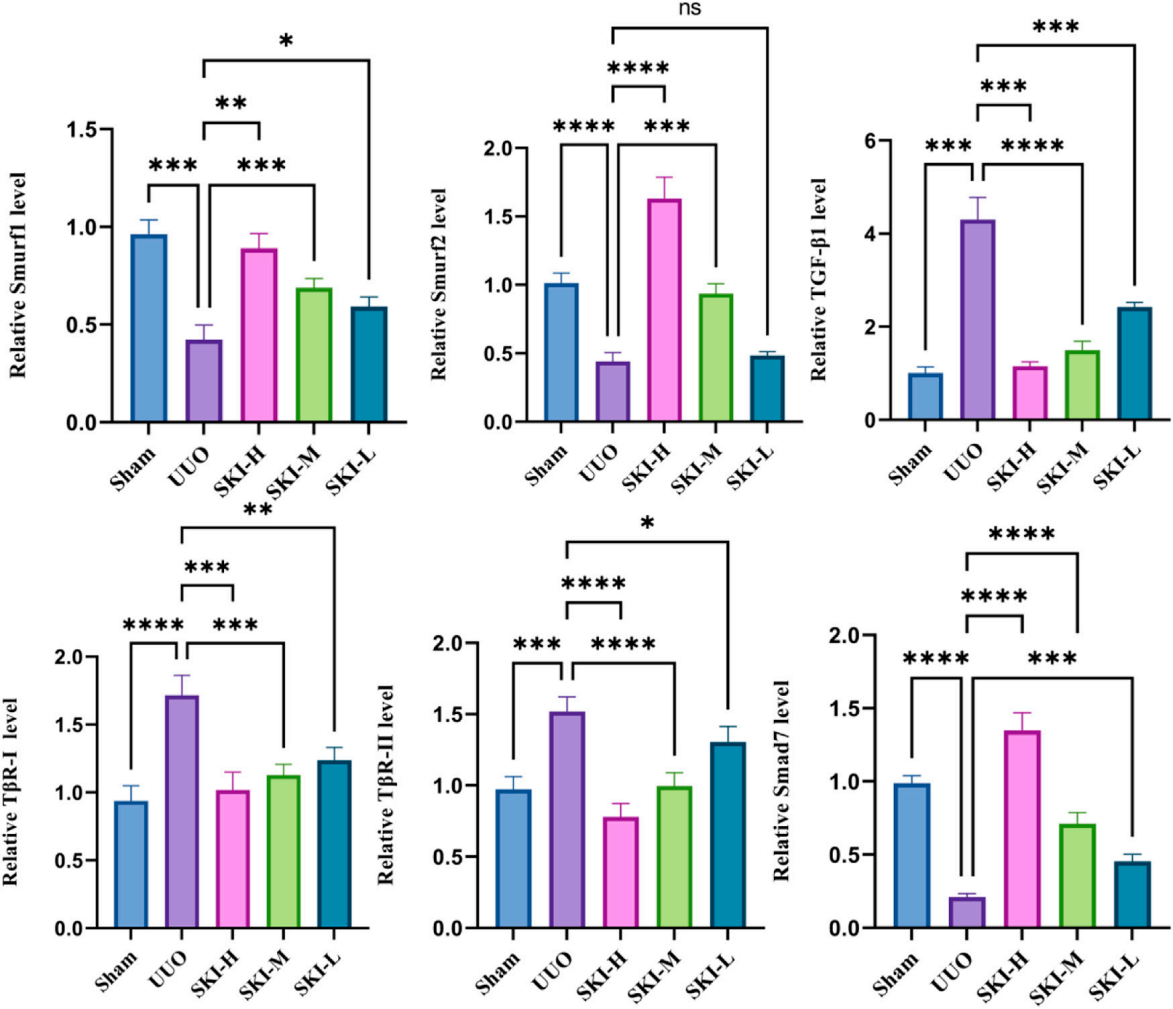
Effect of Shenkang injection on mRNA of TGF-β/Smads signaling pathway. The mRNA expressions of TGF-β1, TβR-I, TβR-II, Smad7, Smurf1, and Smurf2 in renal tissues were detected by RT-PCR.

The signal transduction of TGF-β/Smad was detected by RT-PCR and Western blotting. Compared with the Sham, the UUO significantly upregulated TGF-β1, TβR-I, and TβR-II and downstream Smad3, Smad2, mRNA, and protein levels (*p* < 0.05), and the phosphorylation levels of P-Smad2/3 and P-smad3 increased (*p* < 0.05), while the mRNA and protein levels of Smad7, Smurf1, and Smurf2 decreased (*p* < 0.05), and ubiquitin decreased. Compared with the UUO, the mRNA or protein of TGF-β1, TβR-I, TβR-II, Smad7, Smurf1, and Smurf2 in each treatment group of Shenkang injection showed opposite trends (*p* < 0.01) **(** Figures 5 and 6).

**FIGURE 7:**
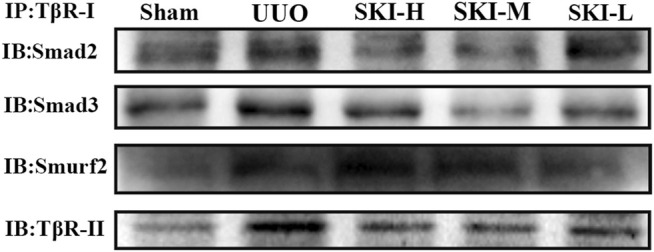
Regulation of ubiquitination by Shenkang injection on TGF-β/Smad signaling pathway.

In order to further study the internal interaction of Shenkang injection on the TGF-β/Smad signaling pathway in UUO model mice, fresh kidney tissues were taken for immunoprecipitation experiments. Compared with the model group, Shenkang injection can inhibit the interaction of TβR-I/Smad2, TβR-I/Smad3, TβR-I/TβR-II, TβR-I/Smurf2, and can also be used in ubiquitination detection. It was found that Shenkang injection can promote the ubiquitination of TβR-I by Smad ubiquitin ligase Smurf2 by promoting the expression of ubiquitination (Figure 7).

### 4.5 Effect of Shenkang Injection on HK-2 Cell Activity

**FIGURE 8:**
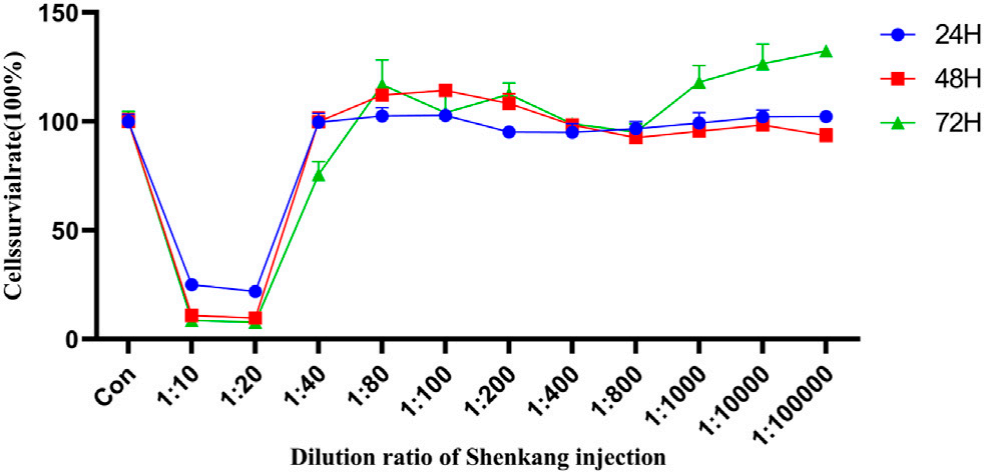
Effect of Shenkang injection with different dilution on HK-2 cell viability.

The cytotoxicity of Shenkang Injection to HK-2 is shown in Figure 8. From the 11 concentrations (1:10, 1:20, 1:40, 1:80, 1:100, 1:200, 1:400, 1:800, 1:1,000, 1:10,000, and 1:100,000), The cell survival rate was observed at 24, 48, and 72 h. At 72 h, The effects of Shenkang injection concentration on the survival rate of HK-2 cells were 14.92%, 13.12%, 91.78%, 110.62%, 107.11%, 105.39%, 97.48%, 104.31%, 109.07%, and 109.46%, respectively. Therefore, 1:40, 1:80, and 1:100 were selected as the high, medium, and low concentrations (SKI-H, SKi-M, and SKi-L) of Shenkang injection.

### 4.6 Shenkang Injection Inhibited the Effects of TGF-β1 on the Expression of EMT Signal Protein and mRNA in HK-2 Cells

**FIGURE 9:**
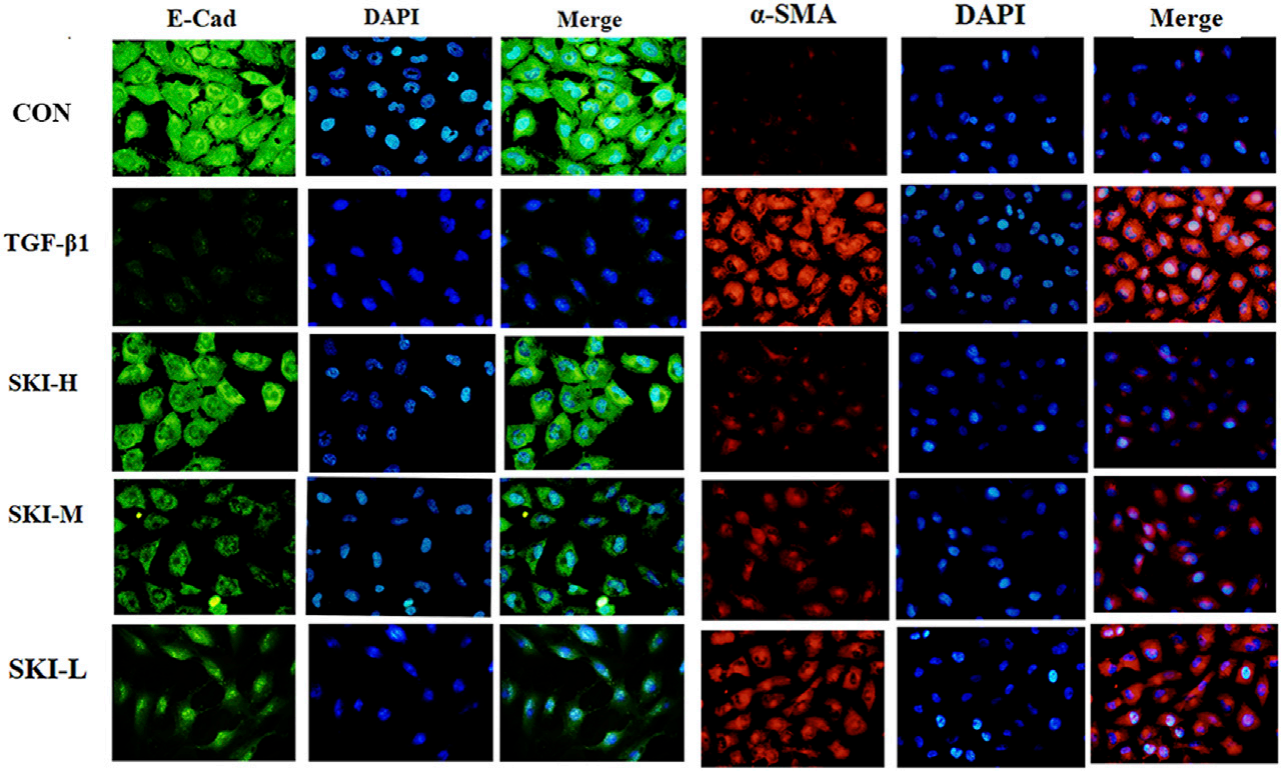
Shenkang injection can inhibit EMT of HK-2 cells. Fluorescence intensity was analyzed with E-Cad (green) and α-SMA (red) antibodies by immunofluorescence method, and nuclei were stained with DAPI (blue). Then the three fluorescence images were merged.

Shenkang injection with a dilution of 1:40, 1:80 and 1:100 was used to intervene in the transdifferentiation process of HK-2 cells induced by TGF-β1. HK-2 cells treated with TGF-β1 reduced the fluorescence intensity of the epithelial cell marker E-cadherin (E-Cad). The fluorescence intensity of α -smooth muscle actin (α-SMA) was increased. Compared with the TGF-β1 group, Shenkang injection could inhibit the EMT effect of HK-2 cells by decreasing the expression of α-SMA protein (*p* < 0.01) and increasing the expression of E-Cad protein (*p* < 0.01), and the morphology of HK-2 cells was also well protected. The inhibitory effect of Shenkang injection on EMT of HK-2 cells was positively correlated with dose, as shown in Figure 9.

### 4.7 Effects of Shenkang Injection on TGF-β1 Induced TGF-β/Smad Signaling Pathway Activation

**FIGURE 10:**
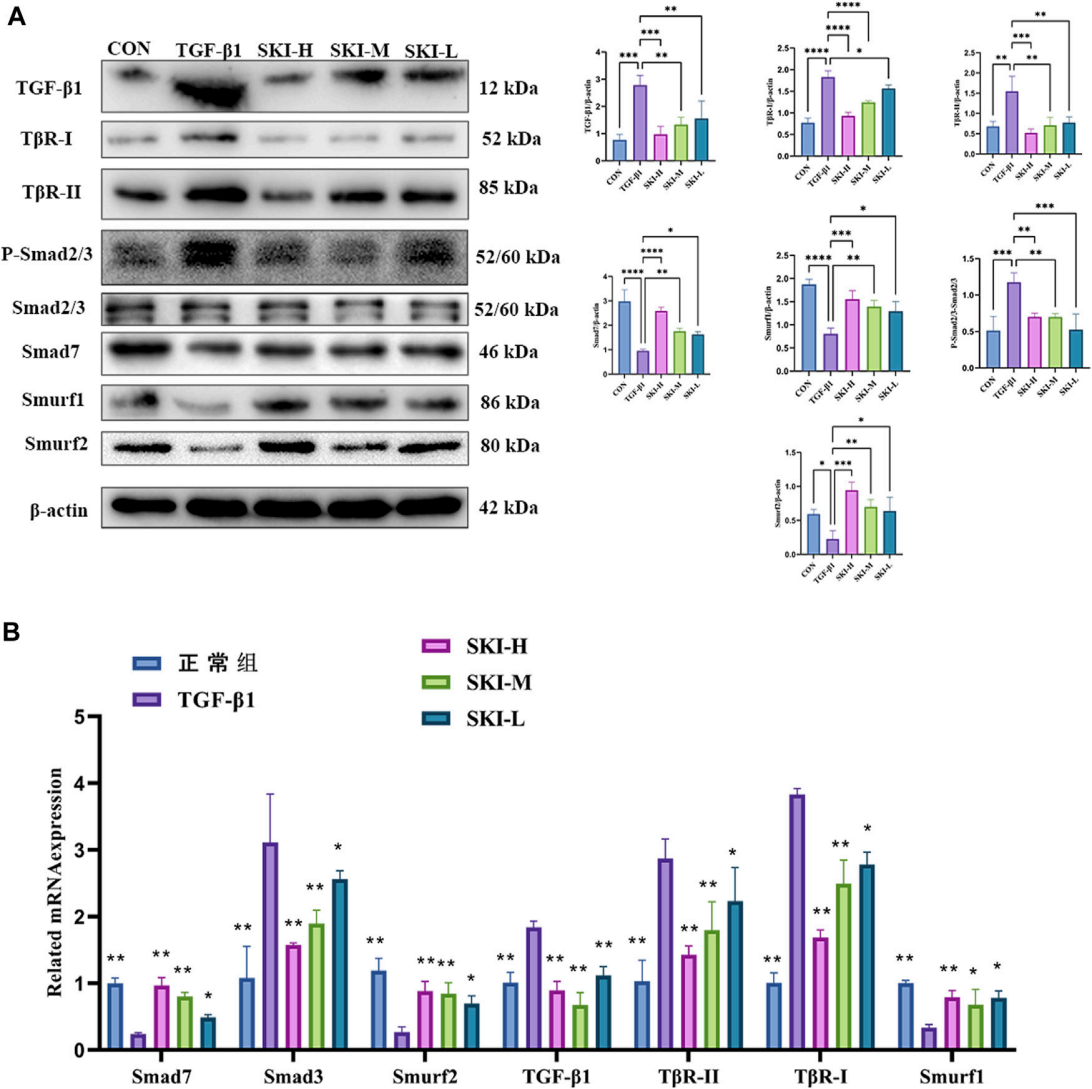
Effects of Shenkang injection on TGF-β/Smads signaling pathway. **(A)** Western blot was used to detect the expression of TGF-β1, TβR-I, TβR-II, P-Smad2/3, SmAD2/3, Smurf1, Smurf2, Smad7, and ubiquitin proteins. **(B)** THE mRNA expressions of TGF-β1, TβR-I, TβR-II, Smad3, Smurf1, Smurf2, and Smad7 were detected by RT-PCR. Data are shown as mean ± SD. *p < 0.05, **p < 0.01, ***p < 0.001, and ****p < 0.0001.

Compared with the TGF-β1 group, Shenkang injection decreased the protein expression of TGF-β1, TβR-I, and TβR-II in HK-2 cells (*p* < 0.01) and decreased the phosphorylation of Smad3 and Smad2/3 (*p* < 0.01). It promoted the expression of Smad7, Smurf1, and Smurf2 proteins (*p* < 0.01) and increased the expression of ubiquitin, suggesting a strong protein ubiquitination reaction in cells (Figure 10A). We examined mRNA levels of TGF-β1, TβR-I, TβR-II, Smad7, Smurf1, Smurf2, and Smad3 in the TGF-β/Smad signaling pathway. Shenkang injection could promote the mRNA expression of Smad7, Smurf1, and Smurf2 in HK-2 cells treated with TGF-β1 (*p* < 0.01). The mRNA expressions of TGF-β1, TβR-I, TβR-II, and Smad3 were decreased (*p* < 0.01) (Figure 10B).

### 4.8 The Effect of Shenkang Injection on the Interaction Among TβR-Ⅰ-TβR-II, TβR-Ⅰ/II-Smad2, TβR-Ⅰ/II-Smad3, and TβR-Ⅰ/II-Smad2/3

**FIGURE 11:**
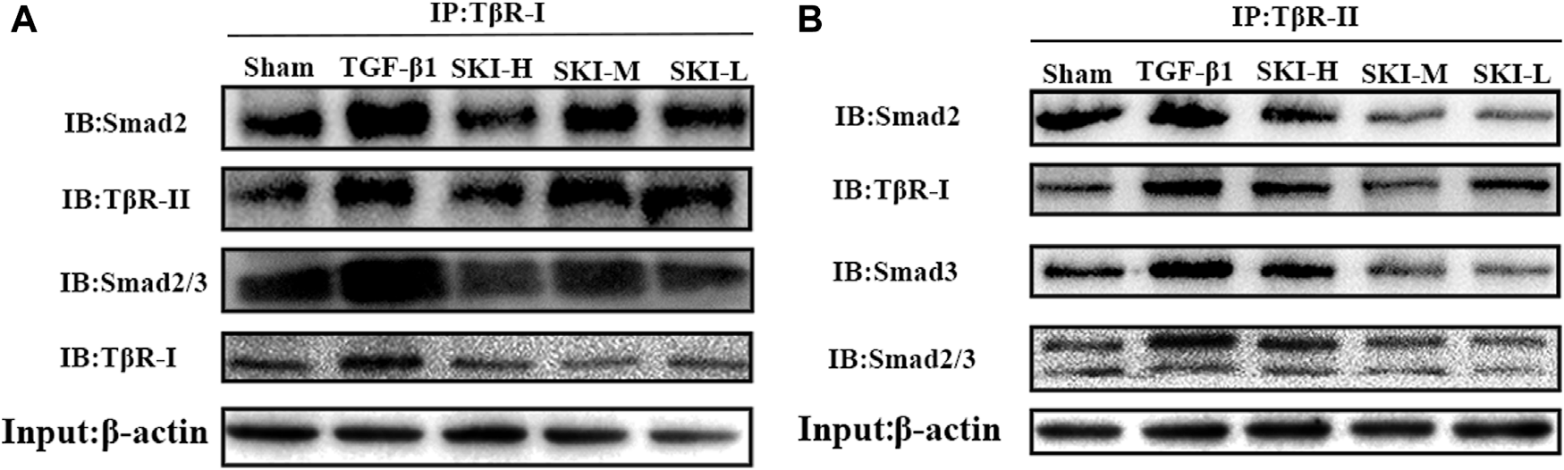
Effects of Shenkang injection on binding of TβR-I/II to Smad2 and Smad3 proteins. **(A)** HK-2 cell lysate was immunoprecipitated (IP) with TβR-I antibody, and then Smad2 and TβR-II, Smad2/3 and TβR-I antibodies performed with to Western blotting (IB). **(B)** HK-2 cell lysate was immunoprecipitated (IP) with TβR-II antibody, and then Smad2,TβR-I, Smad3 and Smad2/3 antibodies performed with to Western blotting (IB).

TβR-Ⅰ-TβR-IⅠ, TβR-Ⅰ/II-Smad2, TβR-Ⅰ/II-Smad3, and TβR-I/II-Smad2/3 of HK-2 cells treated with TGF-β1 were increased compared with normal HK-2 cells. The HK-2 cells treated with Shenkang injection significantly inhibited the binding of TβR-Ⅰ-TβR-IⅠ, TβR-Ⅰ/IISmad2, TβR-Ⅰ/II-Smad3, and TβR-Ⅰ/II- Smad2/3 **(** Figure 11 **)**.

### 4.9 Effects of Shenkang Injection on Smurf1-Smad7, Smurf2-Smad7, Smurf2-Smad2, and Smurf1-TβR-I Proteins in HK-2 Cells Induced by TGF-β1

**FIGURE 12:**
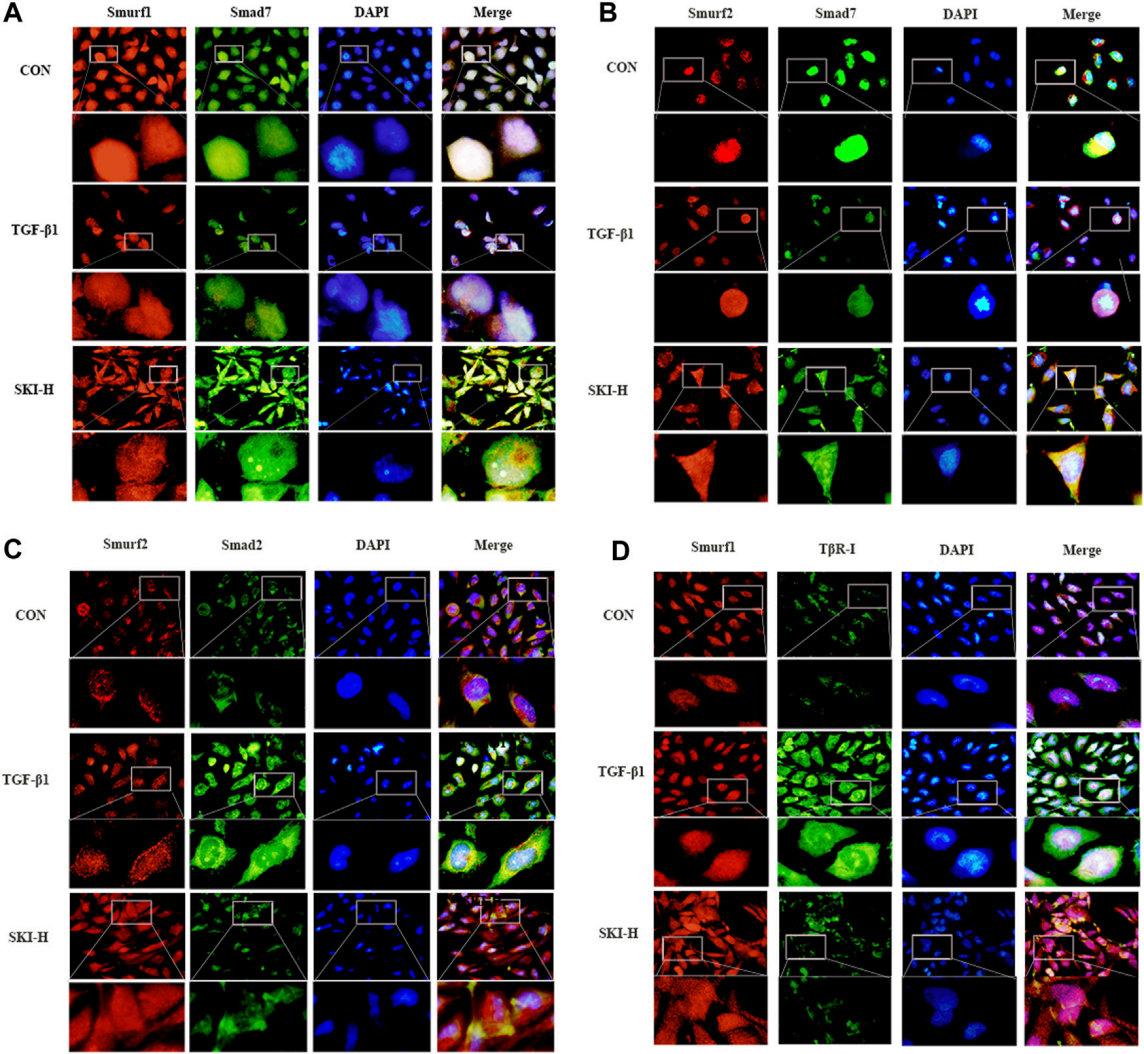
Effects of Shenkang injection on binding and translocation of Smurf1/2 to Smad7, Smad2, and TβR-I. **(A)** Immunofluorescence method was used to detect the localization of Smurf1 (red) and Smad7 (green) proteins in HK-2 cells and the expression of fluorescence intensity and DAPI staining nuclei (blue). **(B)** Immunofluorescence method was used to detect the localization of Smurf2 (red) and Smad7 (green) proteins in HK-2 cells and the expression of fluorescence intensity And DAPI staining nuclei (blue). **(C)** Immunofluorescence method was used to detect the localization of Smurf2 (red) and Smad2 (green) proteins in HK-2 cells and the expression of fluorescence intensity And DAPI staining nuclei (blue). **(D)** The localization of Smurf1 (red) and TβR-I (green) proteins in HK-2 cells and the expression of fluorescence intensity were detected by immunofluorescence, and the nuclei were stained with DAPI (blue).

In order to further verify that Shenkang injection can promote the ubiquitination and degradation of the TGF-β/Smads signaling pathway, we used subcellular localization immunofluorescence technology to observe Smurf1-Smad7, Smurf2-Smad7, Smurf2-Smad2, and Smurf1-TβR-I, respectively. Before and after the translocation of HK-2 cells treated with TGF-β1, it was found that the fluorescence expression of Smad2 and TβR-I in TGF-β1 cells was increased compared with that of HK-2 cells under normal conditions, the fluorescence intensity of Smurf1, Smurf2, and Smad7 is weak, and the expression of Smurf1, Smurf2, and Smad7 is mainly in the nucleus, and the intensity of nuclear translocation is less (Figures 12C and D). Shenkang injection treatment can significantly reduce the fluorescence intensity of Smurf1, Smurf2, and Smad7 in HK-2 cells induced by TGF-β1 and promote Smurf1-Smad7, the nuclear translocation of Smurf2-Smad7 promotes the binding of Smad ubiquitin ligases Smurf2 and Smurf1 to TβR-I and Smad2, and the fluorescence intensity of TβR-I and Smad2 is greatly reduced (Figures 12A and B). This may be related to the promotion of Smurf1-Smad7 and Smurf2-Smad7 nuclear translocation by Shenkang injection.

### 4.10 Effect of Shenkang Injection on Metabolic Spectrum of UUO Mice

#### 4.10.1 Metabolic Profile Analysis

**FIGURE 13:**
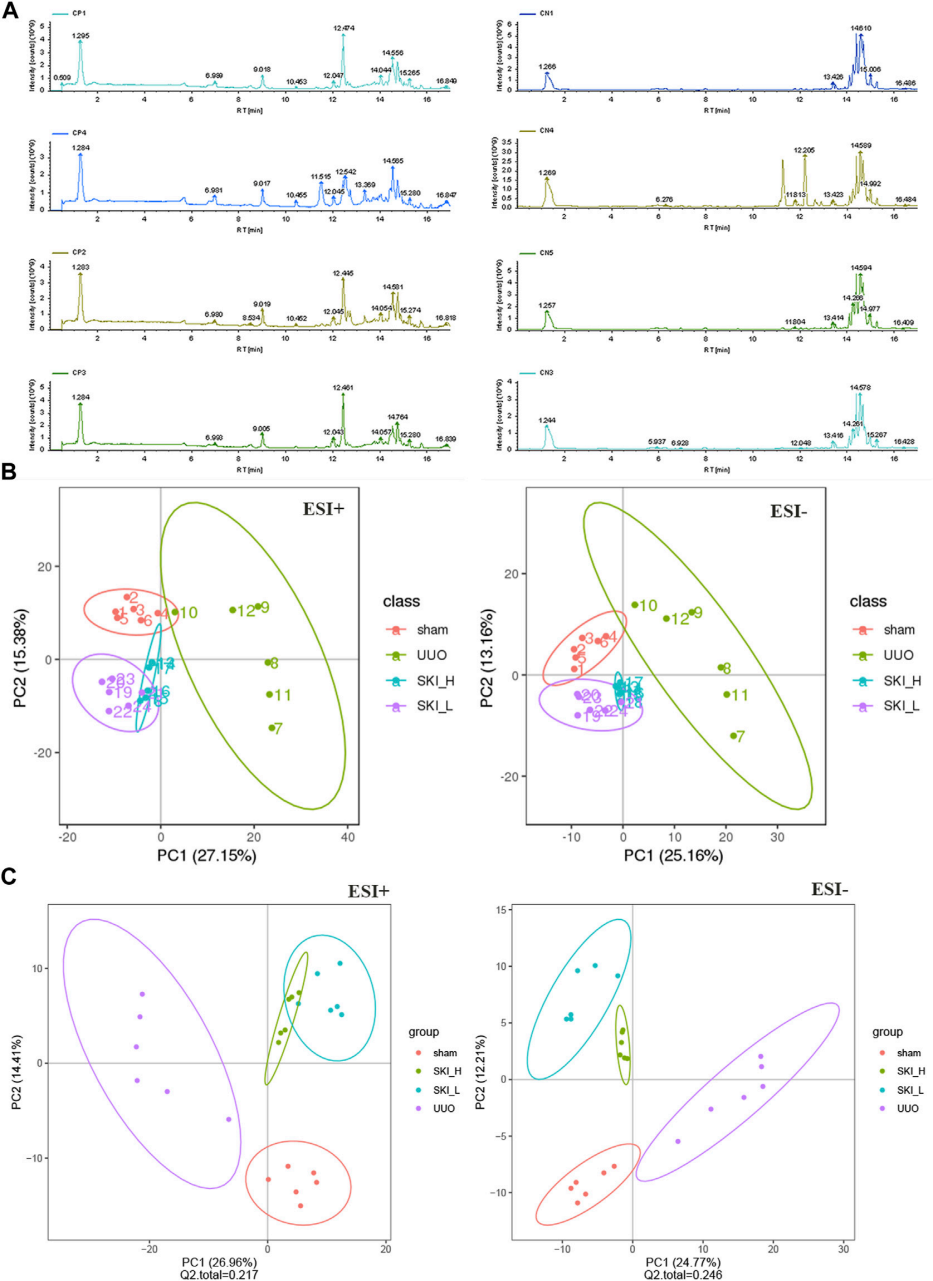
PCA and PLS-DA scores of metabolic differences in Sham, UUO, and SKI group. **(A)** The representative BPI chromatograms (ESI+) of metabolomic samples. **(B)** PCA scores of mice in each group. **(C)** PLS-DA scores of mice in each group. Red dots for Sham; Purple dots for UUO; Blue and green dots for SKI.

Pearson correlation coefficient between QC samples was calculated based on the relative quantitative value of metabolites. The higher the correlation of QC samples (R2 is closer to 1), the better the stability of the whole testing process and the higher the data quality. The correlation of QC samples is shown in Figure 13A. The correlation coefficient between QC samples is close to 1, indicating high correlation. The TIC diagram showed that under positive and negative ion modes, there were certain differences in the intensity of some spectral peaks in the TIC diagram of serum samples in each group, indicating that the metabolic spectra of samples in each group were different to some extent (Figure 13B). The peaks extracted from all experimental samples and QC samples were analyzed by PCA after univariate scaling (univariate normalization). The smaller the difference of QC samples, the better the stability of the whole method and the higher the data quality, which is reflected in the PCA analysis diagram, that is, the distribution of QC samples will gather together. As shown in Figure 13C, QC samples have small differences, good stability, and reliable data quality.

#### 4.10.2 Differential Metabolite Screening Results

**FIGURE 14:**
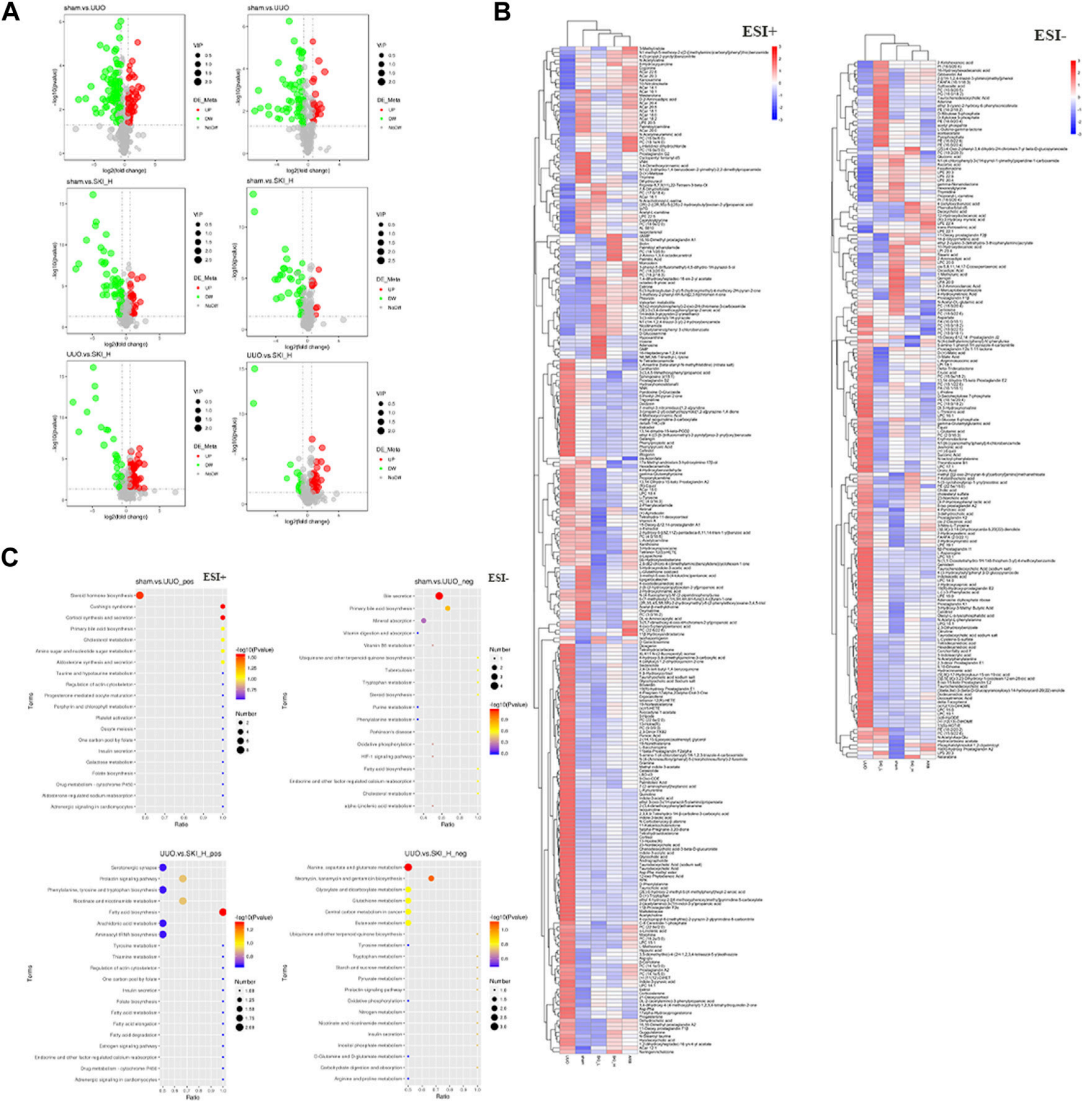
Metabolomic analyses of mice samples from the Sham, UUO and SKI groups. **(A)** Differential metabolite volcano map. Each dot in the volcano map represents a metabolite; the significantly upregulated metabolite is represented by red dots, the significantly downregulated metabolite is represented by green dots, and the size of the dot represents VIP value. **(B)** Differential metabolite cluster heat map. **(C)** KEGG enrichment bubble diagram. Color saturation indicates the metabolite expression value, blue represents the lowest expression, and red represents the highest expression.

The screening of differential metabolites mainly refers to VIP, FC, and *p*-value. VIP refers to the variable projection importance of the first principal component of the PLS-DA model. The thresholds were set as VIP>1.0, FC > 1.5, or FC < 0.667 and *p* < 0.05. The screened differential metabolites are shown in Table 3 and Figures 14A.

**TABLE 3:** Expression of differential metabolites between the UUO model group and the SKI group.

Metabolite	Nucleo plasmic relation m/z	Retention time	P-value	VIP	Domino effect
l-Glutamic acid	147.05	1.20	1.83	0.00	**↑**
Orotic acid	156.02	1.33	2.02	0.00	**↑**
Aspartate	133.04	1.20	2.01	0.00	**↑**
Adenosine diphosphate ribose	559.07	1.17	1.74	0.00	**↑**
Thromboxane B1	354.24	11.93	2.00	0.00	**↑**
Succinic acid	118.03	1.27	1.11	0.04	**↑**
Phenylpyruvic acid	164.05	8.88	1.98	0.00	**↑**
Palmitic acid	273.27	12.65	2.22	0.00	**↑**
l-Tyrosine	181.07	1.37	1.53	0.00	**↑**
α-Linolenic acid	278.22	13.35	1.30	0.00	**↑**
D-(+)-Tryptophan	204.09	9.23	1.94	0.00	**↑**
Sphingosine (d18:1)	299.28	13.63	2.00	0.00	**↑**
Indole-3-acetic acid	175.06	9.69	1.63	0.00	**↑**
Nicotinamide	122.05	1.93	1.67	0.00	**↑**
Xanthosine	284.08	4.99	1.27	0.00	**↑**
Acetylcholine	145.11	1.33	1.89	0.00	**↑**
Methyl indole-3-acetate	189.08	10.45	1.41	0.01	**↑**
l-Kynurenine	208.08	5.24	1.49	0.01	**↑**
23-Nordeoxycholic acid	378.28	11.58	1.44	0.02	**↑**
Taurohyocholic acid sodium salt	537.27	12.68	1.71	0.00	**↑**
Taurocholic acid	515.29	12.59	1.71	0.00	**↑**
Adenosine	267.10	3.10	1.68	0.00	**↑**
Dehydrocholic acid	402.24	12.11	1.29	0.01	**↑**
Inosine	268.08	1.33	1.23	0.03	**↑**
Hypoxanthine	136.04	1.99	1.06	0.04	**↑**
l-Argininosuccinic acid	290.12	1.19	2.08	0.00	**↑**
Orotic acid	156.02	1.33	1.60	0.00	**↑**
Cholic acid	408.29	11.33	1.38	0.00	**↑**
16-Hydroxyhexadecanoic acid	254.22	14.25	1.59	0.00	**↑**
Hexadecanedioic acid	286.21	12.30	1.89	0.00	**↑**
Adenine	135.05	6.12	1.57	0.01	**↑**
Acetoacetate	102.03	1.42	1.43	0.02	**↑**
Taurodeoxycholic acid sodium salt	521.28	11.42	1.25	0.04	**↑**
Citrate	350.21	2.54	1.47	0.01	**↓**
Taurine	132.05	3.27	1.69	0.01	**↓**
Neuraminic acid	283.24	7.36	1.21	0.01	**↓**
Propionyl-l-carnitine	217.13	9.23	1.59	0.02	**↓**
4-(3-Hydroxybutyl)phenyl β-D-glucopyranoside	374.16	10.41	1.25	0.02	**↑**
LPE 20:3	503.30	14.71	1.78	0.02	**↑**
LPE 15:0	439.27	14.49	1.52	0.02	**↑**
Indoleacetic acid	175.06	6.21	1.42	0.02	**↑**
LPC 15:0	541.34	14.40	1.60	0.02	**↑**
2-Hydroxycaproic acid	132.08	3.66	1.46	0.02	**↑**
Fexofenadine	501.29	15.09	1.47	0.03	**↓**
PE (16:0/20:4)	739.52	16.57	1.21	0.03	**↓**
Hexanoylglycine	173.11	8.03	1.52	0.03	**↓**
Erythronolactone	118.03	1.20	1.34	0.03	**↑**
Succinic acid	118.03	1.27	1.11	0.04	**↑**
L-(-)-3-Phenyllactic acid	166.06	6.02	1.17	0.04	**↑**
Equol	242.09	9.40	1.32	0.04	**↑**
5-Amino-1-phenyl-1H-pyrazole-4-carbonitrile	184.07	6.19	1.12	0.04	**↑**
Gluconic acid	196.06	1.22	1.26	0.04	**↓**
trans-Petroselinic acid	564.51	14.71	1.42	0.05	**↓**
8-Iso prostaglandin A2	334.21	12.75	2.01	0.00	**↑**
l-Argininosuccinic acid	290.12	1.19	2.08	0.00	**↑**
Cholesteryl sulfate	466.31	12.66	1.53	0.00	**↑**
D-(+)-Malic acid	134.02	1.14	1.87	0.00	**↑**
D-Malic acid	134.02	1.19	1.66	0.00	**↑**
Sulfoacetic acid	139.98	1.16	1.47	0.00	**↓**
Prostaglandin K2	332.20	10.97	1.18	0.00	**↑**
gamma-Glutamylglutamic acid	276.10	1.15	1.87	0.00	**↑**
Thymidine	242.09	5.73	1.31	0.00	**↓**
Cholic acid	408.29	11.33	1.38	0.00	**↑**
16-Hydroxyhexadecanoic acid	254.22	14.25	1.59	0.00	**↓**
LPI 18:1	598.31	14.21	1.84	0.00	**↑**
LPE 22:1	535.36	15.82	1.49	0.00	**↑**
2-Ketohexanoic acid	130.06	5.58	1.90	0.00	**↓**
D-Ribulose 5-phosphate	194.04	1.19	1.32	0.00	**↓**
L-Gulono-gamma-lactone	178.05	1.29	1.68	0.00	**↓**
Hexadecanedioic acid	286.21	12.30	1.89	0.00	**↑**
Corchorifatty acid F	328.23	10.89	1.83	0.00	**↑**
PI (16:0/20:4)	858.53	16.04	1.59	0.00	**↓**
N-Lactoyl-phenylalanine	237.10	7.95	1.77	0.01	**↑**
Pyrophosphate	177.94	1.19	1.66	0.01	**↓**
5-(3-Cyclohexylprop-1-ynyl)nicotinic acid	243.13	11.38	1.25	0.01	**↑**
23-Norcholic acid	394.27	11.43	1.43	0.01	**↑**
LPE 20:4	501.29	14.50	1.58	0.01	**↑**
Tetradecanedioic acid	258.18	11.32	1.58	0.01	**↑**
PE (18:0/20:4)	767.55	16.13	1.03	0.01	**↓**
PC (16:0e/18:2)	803.61	16.29	1.43	0.01	**↑**
Docosatrienoic acid	334.29	14.94	1.30	0.01	**↑**
Dodecanedioic acid	230.15	9.80	1.58	0.01	**↑**
2,3-Dinor prostaglandin E1	308.20	12.54	1.49	0.01	**↑**
Adenine	135.05	6.12	1.57	0.01	**↓**
PC (16:0/20:5)	839.57	16.16	1.50	0.02	**↓**
l-Cysteine-S-sulfate	200.98	1.21	1.38	0.02	**↑**
8-iso-15-keto Prostaglandin E2	332.20	11.38	1.35	0.02	**↑**
Acetoacetate	102.03	1.42	1.43	0.02	**↓**
11-Deoxy prostaglandin F2β	338.25	12.09	1.06	0.02	**↑**
3-Indoleacrylic acid	187.06	8.20	1.27	0.02	**↑**
Taurochenodeoxycholic acid	499.30	11.40	1.26	0.02	**↑**
(+/-)-Equol	242.09	10.83	1.48	0.02	**↑**
Delta-Tridecalactone	212.18	13.45	1.35	0.03	**↑**
Hydrocinnamic acid	150.07	8.17	1.52	0.03	**↑**
Acetyl phospahte	139.99	1.17	1.19	0.04	**↓**
Dl-P-Hydroxyphenyl lactic acid	182.06	1.35	1.41	0.04	**↑**
Erucic acid	338.32	15.50	1.13	0.04	**↑**
2-Mercaptobenzothiazole	166.99	9.75	1.21	0.04	**↑**
Jasmonic acid	210.13	12.19	1.39	0.04	**↑**
Ethyl3-cyano-2-hydroxy-6-phenylisonicotinate	268.08	3.61	1.16	0.04	**↓**

Statistical significance levels were determined by the ANOVA test. Only metabolites with *p*-values of less than 0.05 were deemed to be statistically significant.

There were more differential metabolites in the UUO group and the Sham group, and the differential metabolites showed a trend of decrease in the UUO mice after drug intervention. As shown in Table 3, in this study, VIP > 1 and *p* < 0.05 were used as the screening basis. A total of 103 kinds of differential metabolites were screened out. Compared with the UUO group, 68 substances in the administration group were upregulated, and 35 substances were downregulated.

Cluster analysis was used to judge the metabolic patterns of metabolites under different experimental conditions. Metabolites with similar metabolic patterns may have similar functions or participate in the same metabolic process or cellular pathway. Therefore, by clustering metabolites with the same or similar metabolic patterns, the functions of certain metabolites can be inferred. As shown in Figure 14B, blue and red separation was obvious between the Sham and the UUO, indicating a significant difference in metabolites. The color of the Sham was similar to that of the drug group, and the distribution of red and blue was similar, indicating that the metabolites of the drug group changed in the direction of the Sham.

Based on the abovementioned analysis, we used metabolic pathway analysis to reveal the internal relationships between matched metabolites. The result is shown in Figure 14C. The key pathways involved in Shenkang injection on serum metabolites of UUO mice are the biliary secretion pathway, metabolism of taurine and low taurine, steroid metabolism, phenylalanine metabolism, phosphoinositol metabolism, purine metabolism, steroid hormone biosynthesis, amino acid metabolism, carbohydrate digestion and absorption. α -linolenic acid metabolism, and citric acid cycle (TCA cycle) pathway.

## 5 Discussion

Renal fibrosis is a very complex and irreversible pathological process that involves the activation and mutual interference of multiple pro-fibrotic signaling pathways and is a late-stage feature of all types of chronic kidney disease (CKD) (Meng et al., 2014). TGF-/Smads signaling is a powerful fibrogenesis pathway (Meng et al., 2016). Activation of TGF-β/Smads signals leads to extracellular matrix synthesis and deposition, podiocyte depletion, mesangial dilation, renal tubular epithelial fibrosis transformation, and myoblast fibroblast activation (Chen et al., 2018). Elevated levels of renal tubules TβR-I and TβR-II were found in the kidneys of unilateral ureteral obstruction (UUO) model mice. In the renal tubules of mice, the overexpression of TβR-I leads to increased renal oxidative stress and inflammatory cell infiltration, recreating the phenotype of renal fibrosis. It has been reported that Smads ubiquitin ligase SMurF1/2 plays a negative regulatory role by promoting ubiquitination and degradation of upstream proteins of the TGF-β/Smad pathway (Inoue and Imamura, 2008). Smurfs have been identified as modulators of the TGF-β/Smad signaling pathway, interacting with Smad2 and TβR-I and mediating ubiquitin-mediated degradation of these signaling components (Zhang et al., 2001). According to these findings, the renal tubules are the primary targets of TGF-/Smads signaling in renal fibrosis. Previous research has shown that Shenkang injection can negatively regulate TGF-/Smads signaling by lowering TGF- and P-smad3 expression in 5/6 nephrectomized rats, thereby delaying the progression of renal fibrosis and EMT of renal tubular epithelial cells (Wu et al., 2015). Luo et al. (2021) showed that SKI and its components (including chrysophenol, emoflavin, and emoflavic acid) can target the IƙB/NF-ƙB and Keap1/Nrf2 (Chen et al., 2019) signaling pathways to inhibit oxidative stress and inflammation, thereby preventing renal fibrosis. The Janus kinase/signal transduction and transcriptional activator (JAK/STAT) pathway is a multipotent signal cascade of multiple growth factors and cytokines, and activation of the JAK/STAT pathway is increased in renal interstitial fibroblasts induced by unilateral ureteral obstruction (UUO) (Darnell et al., 1994). Catalase reducing protein 5 (Prdx5) (Choi et al., 2016), cytokine signaling protein 1 (SOCS1), and SOCS3 (Ogata et al., 2006) are positive regulators of this pathway. Qin et al. (2021) found that Shenkang injection can negatively regulate the JAK2/STAT3 signaling pathway by inhibiting SOCS and Prdx5 proteins and can effectively inhibit renal fibroblast activation and renal fibrosis in UUO mice. Other studies have shown that Shenkang injection can alleviate CKD and renal fibrosis by regulating anti-aging molecules (Fu et al., 2019), er stress-induced apoptosis (Wang et al., 2021), and the AURKB/RacGAP1/RhoA pathway (Wang et al., 2022).

Similar to previous studies, the Scr and BUN levels in UUO mice were higher than those in the Sham, but not significantly higher, considering the chronic stage of kidney injury on day 14 of modeling; It has been reported that the increased levels of Scr and BUN in UUO mice were more obvious on the 7th day than on the 14th day, which may indicate that the body was in the stage of acute injury on the 7th day (Xu et al., 2014). During EMT, renal tubular epithelial cells acquire a mesenchymal phenotype, with enhanced migration capacity and increased extracellular mechanisms due to cytoskeletal changes (Kalluri and Weinberg, 2009; Li YK. et al., 2018). In our study, HE and Masson staining showed severe renal structural damage in UUO mice, including decreased number of renal tubules, coexisting dilatation and atrophy of renal tubules, apoptosis of tubular epithelial cells, hydronephrosis of the kidneys, and renal interstitial fibrosis, which were similar to those reported in the literature. In the Shenkang injection high, medium, and low concentration groups, the abovementioned symptoms were relieved and recovered to varying degrees. Due to the presence and proliferation of fibroblasts, the positive expressions of collagen I and collagen III, α-SMA, and laminin in the renal interstitium of UUO mice were observed in immunohistochemical sections, while the disappearance of E-Cad protein indicated an increase in the extracellular matrix of the kidney, renal tubular epithelium. The protein skeleton of the cells changed, and the renal tubular epithelial cells blocked the changes of adhesion molecules, suggesting that the epithelial cells were gradually completing the process of EMT. Similar to Masson staining, the basement membrane and space of renal tubules were increased, and the structures of proximal and distal tubules were obviously disordered. These manifestations in animal experiments matched those seen in TGF-1-induced EMT progression of HK-2 cells. TGF-β1 can induce morphological changes and enhance the migration ability of HK-2 cells. After TGF-β1 treatment, mRNA and protein expression of TGF-β1, α-SMA, TβR-I, TβR-II, and Col-I of HK-2 cells were increased. TGF-β-activated signals play a critical role in mediating EMT (Kahata et al., 2018). TGF-β1 induces cell transdifferentiation mechanisms that are very complex, including Smad, MAPK, PI3K, and other pathways. Smad signals (including Smad2, Smad3, and Smad4) are primarily responsible for TGF-β1-induced fibrosis (Massagué and Wotton, 2000; Zhou et al., 2017).

The mRNA and protein levels of Smad3 and Smad2 were increased in UUO mice, and TGF-β1, Tβ r-I, TβR-I, P-Smad3, and P-Smad2/3 increased significantly, which was consistent with the findings of other authors (Honma et al., 2016; Martínez-Klimova et al., 2019). Compared with the UUO group, the Shenkang injection group can reduce the expression of TGF-β1, p-smad3 protein, and mRNA in the obstructed kidney, which is consistent with the results obtained by other researchers using Shenkang injection (Wu et al., 2015). Previous studies have shown that activation of the TGF-β/Smad signaling pathway requires the binding of activated TGF-β1 to TβR-II to activate TβR-I kinases that phosphorylate Smad2 and Smad3. To activate the TGF-/Smads signaling pathway, phosphorylated Smad2 and Smad3 bind to Smad4 to form the Smad complex, which translocates into the nucleus and regulates the transcription of target genes (Lan, 2012; Meng et al., 2016). In our study, we discovered that Shenkang injection decreased the phosphorylation of TGF- type I and type II receptors in the TGF-/Smad signaling pathway, increased the expression of negative regulatory protein Smad7, and promoted the ubiquitination expression of the obstructed kidney. This may be related to the Smad ubiquitin ligases Smurf1 and Smurf2. It has been reported that Smurf1 and Smurf2 selectively interact with TGF-β1 receptors and Smads, preferentially targeting TβR-I and Smad2 (Lin et al., 2000; Ebisawa et al., 2001; Suzuki et al., 2002). In further studies, we found that Shenkang injection can promote the expression of Smurf1 and Smurf2 proteins and mRNA, and the high expression of Smurf1 and Smurf2 can bind to Smad7. Under the action of cohesion protein Smad7, Smad7 transfers Smurf1 and Smurf2 together to the plasma membrane and binds with TβR-I and Smad2 to promote their ubiquitination and degradation. The plasma membrane localization of Smad7 depends on the C2 domain of Smurf1 and the N domain of Smad7 (Suzuki et al., 2002; Tajima et al., 2003). Immunoprecipitation (CO-IP) assay showed that Shenkang injection attenuated the interaction of Smad2-TβR-I, Smad3-TβR-I, and TβR-II-TβR-I, and the binding of Smurf2 to TβR-I was stronger than that of UUO group. *In vitro* cell studies, subcellular localization and immunoprecipitation experiments were used to verify the effect of Shenkang injection on the TGF-β/Smads signaling pathway. TGF-β 1-induced HK-2 cells exhibited stable TR-II/TR-I, TR-I/Smad2, and TR-I/Smad3 protein binding, as well as high fluorescence intensity of Smad2 and TR-I. The fluorescence intensity of Smad7, Smurf1, and Smurf2 proteins was weak (mainly expressed in the nucleus), which was similar to the study by Liu et al. (2019). Shenkang injection can translocate Smurf1-Smad7 and Smurf2-Smad7 into the cytoplasm by promoting the expression and nuclear translocation of Smad7, Smurf1, and Smurf2 proteins, increasing the expression of Smurf1-Smad7 and SmurF2-Smad7 in the cytoplasm. The expression of Smad2 and TβR-I in the cytoplasm was decreased, and the nuclear translocation of Smad2 was inhibited. In immunoprecipitation experiments, Shenkang injection inhibited the binding of TβR-II/TβR-I, TβR-I/Smad2, and TβR-I/Smad3, thus preventing the phosphorylation of Smad2 and Smad3. As a result, the negative effects of Shenkang injection on the TGF-/Smads signaling pathway may be linked to a series of ubiquitination processes in this pathway.

Renal fibrosis is a common pathway of progressive kidney disease, and its pathogenesis is complex and eventually develops into chronic renal failure (CRF). CRF is mainly characterized by a significant decline in renal function, endocrine imbalance, and metabolic disorder (Drawz and Rahman, 2015). From previous studies, we can find that patients with chronic kidney disease have intestinal flora disorders and metabolic products imbalances, leading to malnutrition, anemia, and toxin residues (accumulation of creatinine and urea nitrogen, increased uric acid, metabolic acidosis, etc.) (Anders et al., 2013; Vaziri et al., 2013; Andersen et al., 2017). Therefore, we should prevent or intervene in chronic kidney disease before it develops to the point that CRF is irreversible, which will be beneficial in improving our efficacy and quality of life. With the development of science, more detection methods can help us advance the trend and cognition of diseases. The inherent sensitivity of metabolomics can help us detect subtle changes in biological metabolic pathways so as to help us deeply understand and judge the potential mechanisms of various physiological conditions and abnormal processes (Johnson et al., 2016; Schrimpe-Rutledge et al., 2016).

According to our metabolomics data, 516 and 368 different metabolites were obtained in positive and negative ion mode, respectively. After a series of screening and validation, 100 different metabolites were identified, which helped us to identify and summarize the metabolites of UUO model mice, normal mice, and mice after modeling and administration. These data reflected the imbalance and recovery of metabolites in mice after treatment, including steroid metabolism, taurine and low taurine metabolism, bile acid metabolism, amino acid metabolism, purine metabolism, fatty acid metabolism, and TCA cycle (Figure 13 and Table 3).

Lipids can be divided into eight types: fatty acids, glycolipids, glycerolipids, sterols, polyketones, glycerols, sphingolipids, and enolipids (Zhao et al., 2015). Glycerophospholipid, as a basic component of the cell membrane, plays an important role in signaling pathways and material transport (Hermansson et al., 2011). The level of glycerophospholipid in patients with CKD at different stages also showed a trend of gradual increase, and there was an inverse relationship between glycerophospholipid and glomerular filtration rate in patients with CKD (Zhao et al., 2015; Barrios et al., 2016; Ye and Mao, 2016). The increase of acetate in serum was a marker of sustained renal medulla injury in the UUO model, indicating that renal fibrosis was in a persistent state (Hanifa et al., 2019). We found that the expression of acetate decreased in shenkang injection treatment, which was consistent with the results of our study. After shenkang injection intervention, the expression of ECM markers collagen I and collagen IV decreased in mouse kidneys, and the markers E-Cad and α-SMA of EMT developed in a good direction. The content of fatty acids in the serum of patients with renal disease is higher than that of healthy people. Moreover, the oxidants and key enzymes of fatty acids in patients with renal interstitial fibrosis and mice are lower, resulting in a serious accumulation of cell lipids. Neutrophil release is stimulated by significant increases in leukotriene and 5-hydroxy-eicosapentaenoic acid (5-HETE), 9-HETE, and 15-HETE (Huang et al., 2012; Wang et al., 2016). In our study, we found that the serum levels of fatty acids (tetradecanoic acid, octadecanamide, stearic acid, oleic acid, linoleic acid, palmitamide, palmitic acid, leukotriene E3, leukotriene B, 5-HETE, 9- HETE, 15- HETE, and creatinine) in UUO mice were significantly increased. This result is consistent with the above report; the serum fatty acid metabolites in the Shenkang injection group returned to normal.

Under normal physiological conditions, most of the amino acids are catabolized by various pathways of the kidney (such as asparagase, glutamate dehydrogenase, glutaminase, aminotransferase, and D-AA oxidase), and the amino acids filtered by the kidney are almost completely reabsorbed into the blood by the near convoluted tubules (Wang et al., 2013; Li R. et al., 2018). Renal dysfunction can alter the pathway of amino acid metabolism, altering the homeostasis of the entire amino acid and causing health issues (Li et al., 2020). In our study, amino acid metabolism was disrupted in UUO mice, including histamine, l-glutamic acid, L-aspartate, phenylalanine, L-asparagine, arginine succinate, succinic acid, tryptophan, and 3-methylhistidine. An earlier study showed that serum aspartic acid levels were significantly elevated before and after hemodialysis in patients with end-stage renal disease compared with controls. Compared to all dialysis patients, valine/glycine and tyrosine/phenylalanine ratios were significantly reduced and can be used as biomarkers to assess the risk or presence of kidney disease (Chuang et al., 2006). Shenkang injection can prevent and cure the increase of L-histidine, 3-methylhistidine, uric acid, and glutamine in UUO mice. These findings illustrate the disruption of amino acid metabolic pathways in the UUO model. However, ShenKang injection could promote the recovery of tryptophan, aspartic acid, indole, 3-methyldioxy indole, indoleacetic acid, histamine, l-glutamic acid, l-aspartic acid, phenylalanine, L-asparagine, arginine succinic acid, succinic acid, and 3-methylhistidine in the serum of UUO mice.

Taurine is a product of cysteamine dioxygenase in the metabolic pathway of taurine and pentaurine and has many important functions. It plays the role of an antioxidant and protective agent in the body, promoting the transport of various ions with antioxidant and anti-inflammatory properties (Poljšak and Fink, 2014). Renal fibrosis is a pathological condition that requires the activation and interaction of many pro-fibrotic signaling pathways. It is a late-stage symptom of all forms of chronic kidney disease (CKD), which affects more than 10% of the global population and is a serious public health threat (Bergström et al., 1989). The decrease in taurine observed in our UUO model is consistent with the reported increase in serum taurine in mice treated with Shenkang injection in patients with advanced CKD. The serum levels of adenine, hypoxanthine, guanosine monophosphate, inosine, adenosine diphosphate ribose, xanthine, and canine uric acid in the UUO group were significantly increased, suggesting purine metabolism disorder. Citric acid and isocitrate are intermediates of the tricarboxylic acid cycle. Studies have shown that citric acid can significantly shorten the time of urinary retention, improve renal function indicators, blood biochemical indicators, and inflammatory indicators, maintain internal environmental stability, and reduce the risk of bleeding (Posada-Ayala et al., 2014). In this study, it was found that compared with normal mice, the citric acid content in the model group decreased, resulting in a decrease in the synthesis of its downstream product, isocitrate. Therefore, in the UUO group with a low citric acid content, urine retention was higher and renal cysts were more obvious. The content of citric acid in the Shenkang injection group was higher than that in the UUO group. In conclusion, Shenkang injection can increase the content of taurine in UUO mice, regulate the disorder of purine metabolism and reduce the level of uric acid.

Lipid metabolism disorders have been linked to the TGF-/Smad signaling pathway, according to research. TGF-β-signaling in hepatocytes has been shown to promote hepatic steatosis, stellate cell activation, and fibrosis. TGF-β signal transduction also affects lipid metabolism by regulating the expression of genes involved in fat formation and fatty acid oxidation (Yang et al., 2014). Inhibition of the TGF-β/Smads pathway eliminates changes in gene expression associated with lipid metabolism (Yadav et al., 2011). Yalamanchi et al. (2004) showed that lactic acid significantly increased the activity of TGF-β peptide (TGF-β1, TGF-β2, and TGF-β3), TGF-β receptor (R1, R2, and R3) and TGF-β function, and promoted the increase of collagen and scar tissue, and it provides a good environment for cell transdifferentiation. Zhao et al. (2014) observed upregulated expression of extracellular matrix (ECM) components TGF-β1, connective tissue growth factor (CTGF), fibroblast growth factor (bFGF), and collagen I in an adenine-induced CKD rat model, accompanied by purine metabolism, lipid metabolism, and amino acid metabolism disorder. Cysteine dioxygenase (Cdo) is a well-known key regulator of taurine synthesis. IL-1β, TNF-α, and TGF-β can downregulate the mRNA level of Cdo (Hu et al., 2018). Therefore, the transmission of the TGF-β/Smad signal pathway is closely related to the disorder of lipid metabolism pathway, amino acid metabolism pathway, and purine metabolism pathway, and the regulation of TGF-β/Smad signal transduction can improve the level of disordered metabolites.

**FIGURE 15:**
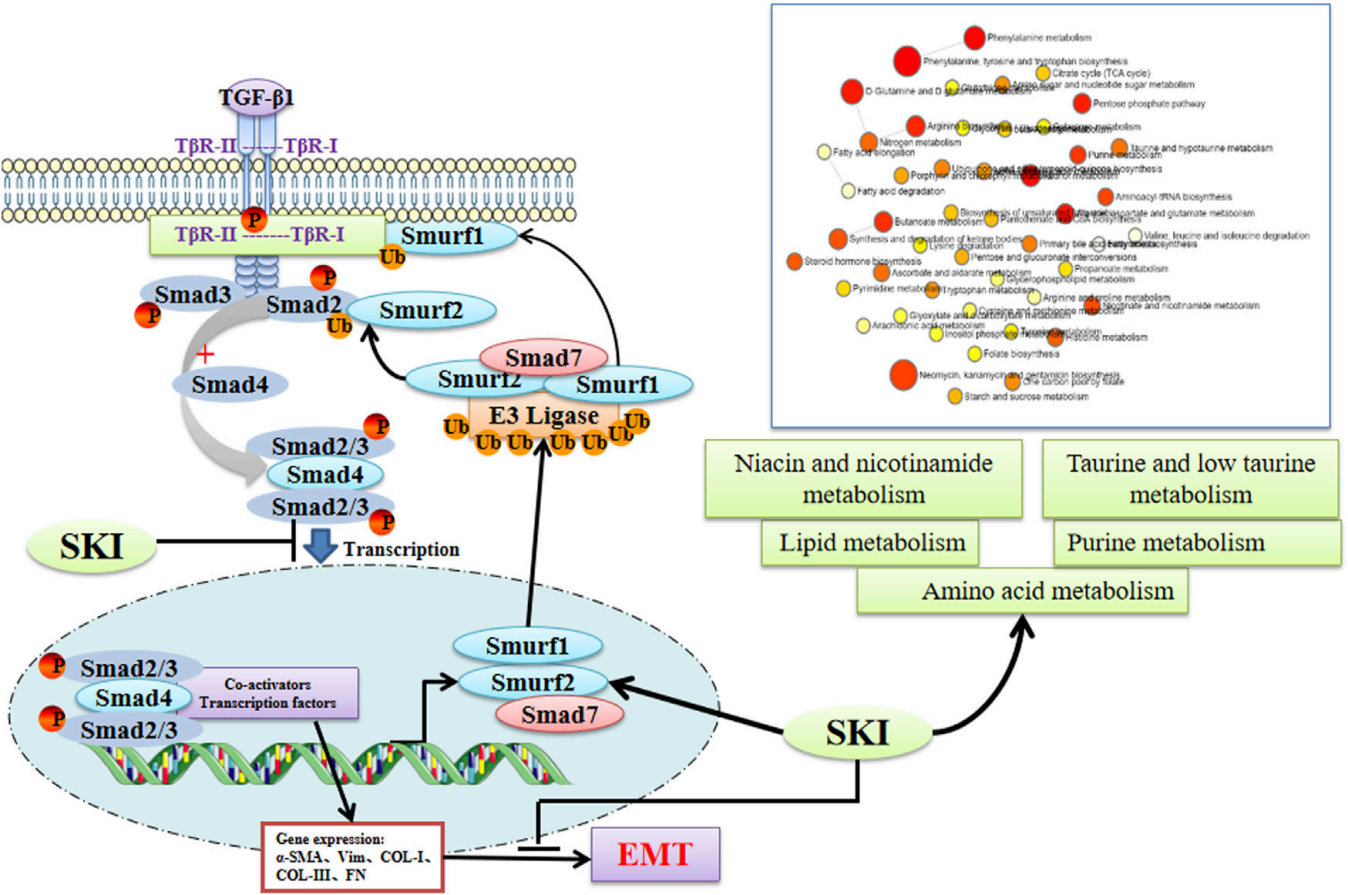
The underlying mechanism of Losartan on the TGF-β/Smad pathway and the metabolomics in UUO mice. SKI can promote the expression of Smad7 and E3 ubiquitin ligases Smurf1 and Smurf2, promote the ubiquitination and degradation of the TGF-β/Smad signaling pathway, and improve the chances of lipid metabolism and amino acid metabolites, so as to achieve the prevention and treatment of renal fibrosis (created with BioRender.com).

In summary, as depicted in Figure 15, our data suggest that the UUO model significantly affects the disruption of various metabolites and the activation of the TGF-β/Smads signaling pathway. Shenkang injection partially reversed the damage caused by the UUO model, so shenkang injection is expected to be an effective drug in the treatment of kidney diseases.

## Data Availability

The original contributions presented in the study are included in the article/Supplementary Material; further inquiries can be directed to the corresponding authors.

## Abbreviations

CKD, chronic kidney disease; TGF-β, transforming growth factor-β; ECM, extracellular matrix; EMT, epithelial–mesenchymal transition; TβRI, TGF-β receptor I; TβRII, TGF-β receptor II; Smurf2, Smad ubiquitin regulatory factor 2; Smurf1, Smad ubiquitin regulatory factor 1; Scr, serum creatinine; BUN, blood urea nitrogen; AKI, acute kidney Injury; Vim, laminin; α-SMA, alpha-smooth muscle actin; UUO, unilateral ureteral obstruction; PC, phosphatidylcholine; PE, phosphatidylethanolamine; LPE, lysophosphatidylethanolamine; LPC, lysophosphatidylcholine; LPA, lysophosphatidic acid; PA, phosphatidic acid; FFA, free fatty acids; CDCA, chenodeoxycholic acid.
