# Supplementary material for: Shenkang Injection for Treating Renal Fibrosis-Metabonomics and Regulation of E3 Ubiquitin Ligase Smurfs on TGF-β/Smads Signal Transduction
Source: Front Pharmacol. 2022 Jun 2;13:849832. doi: 10.3389/fphar.2022.849832 (PMC9201572; doi:10.3389/fphar.2022.849832)
Supplement: Supplementary file 4 [file Table9.DOCX]

All original microscope images in the article are stored in the following link.

Link: https://www.jianguoyun.com/p/DZ_wIS4Q2a_7CRi-3KcE

Access password: V45nTK
